# The genome of the naturally evolved obesity-prone Ossabaw miniature pig

**DOI:** 10.1016/j.isci.2021.103081

**Published:** 2021-09-03

**Authors:** Yaolei Zhang, Guangyi Fan, Xin Liu, Kerstin Skovgaard, Michael Sturek, Peter M.H. Heegaard

**Affiliations:** 1 Translational Immunology Group, Department of Biotechnology and Biomedicine, Technical University of Denmark, Lyngby, Denmark; 2 BGI-Qingdao, BGI-Shenzhen, Qingdao 266555, China; 3 BGI-Shenzhen, Shenzhen 518083, China; 4 China National GeneBank, BGI-Shenzhen, Shenzhen 518120, China; 5 Department of Anatomy, Cell Biology, & Physiology, Indiana University School of Medicine, Indianapolis, IN 46202, USA; 6 Innate Immunology Group, Department of Health Technology, Technical University of Denmark, Lyngby, Denmark

**Keywords:** Physiology, Genetics, Phylogenetics, Animal Physiology

## Abstract

The feral pigs of Ossabaw Island (USA) have an outstanding propensity to obesity and develop complete metabolic syndrome (MetS) upon prolonged high energy dieting. We now report the first high quality genome of the Ossabaw pig with Contig N50 of ∼6.03 Mb, significantly higher than most other published pig genomes. Genomic comparison to Duroc reveals that variations including SNPs, INDELs and one ∼2 Mb inversion identified in Ossabaw pig may be related to its “thrifty” phenotype. Finally, an important positively selected gene (PSG) was found to be *LEPR* (leptin receptor) containing two positively selected sites which may lead to pseudogenization of this gene with possible significant effects on obesity and inflammation development. This work provides the first complete mapping of a genome representing a naturally ‘feast and famine’ evolved phenotype of MetS, serving as a blueprint to guide the search for new targets and new biomarkers for obesity comorbidities.

## Highlights

• The first high quality genome of the Ossabaw miniature pig
• Found important heterozygous genes in the Ossabaw genome
• Identified mutations of obesity-related genes
• Defined candidate targets and biomarkers for obesity comorbidities

## Graphical abstract

**Figure:**
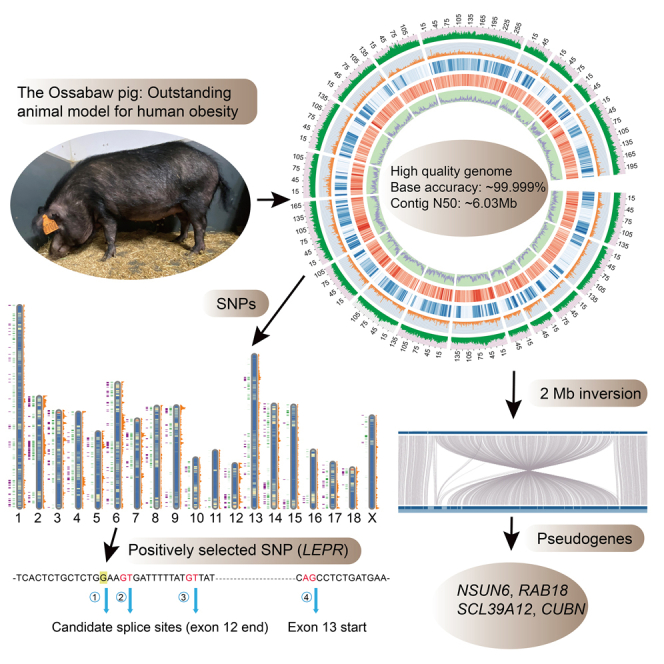

## Introduction

Obesity is a major risk factor for globally prevalent serious diseases like cardiovascular diseases, type 2 diabetes, and cancer, which are leading causes of mortality worldwide (Bluher, 2019). Metabolic syndrome (MetS) is a cluster of risk factors accompanying metabolically unhealthy obesity and includes dyslipidemia, hypertension, insulin resistance and glucose intolerance (O'Neill and O'Driscoll, 2015). There is a great need to understand the complexities of obesity and its associations with disease to form the basis of developing diagnostic tools and interventions to treat and/or prevent obesity and maintain a healthy body weight (Bluher, 2019). To achieve this, animal models faithfully mimicking the complex human state of obesity are pivotal, i.e., human translational animal models in which a complete MetS and development of co-morbidities are obtained upon diet-induced obesity (Kleinert et al., 2018; Reilly and Saltiel, 2017). In this respect, the pig has emerged as highly promising (Bellinger et al., 2006; Kleinert et al., 2018; Spurlock and Gabler, 2008; Zhang and Lerman, 2016). Generally for complex and systemic diseases, pigs are superior to rodent models, as they are much more similar to humans with respect to e.g., the cardiovascular system, inflammation phenotypes and mechanisms, and organ sizes (Zettler et al., 2020). Moreover, certain types of pigs have a phenomenal propensity to obesity and this is especially the case for the Ossabaw pig, which is an outstanding human translatable obesity model (Sturek et al., 2020). Obesity in the Ossabaw pig is reproducibly and rapidly obtained by high energy high fat dieting, presenting as robust MetS (Neeb et al., 2010; Sturek et al., 2020), closely resembling the state observed in metabolically unhealthy obese humans (O'Neill and O'Driscoll, 2015). Notably, the Ossabaw pig is a feral pig breed developed in isolation for approximately 500 years by natural evolution (Sturek et al., 2015) on Ossabaw Island (Georgia, USA), host to the only wild-living colony in the world. They descend from pigs introduced by Spanish explorers around 1500 (Mayer and Brisbin, 1991). The Ossabaw pig is small (30–70 kg adult weight), has the ability to tolerate high salt concentrations in food and drinking water, and is unique among pigs and other ungulates in having the highest adipose reserves of any terrestrial mammal (Brisbin and Mayer, 2001; Stribling et al., 1984). Interestingly, the Ossabaw phenotype results from a ‘thrifty genotype’ characterized by an increased ability to convert food into body fat (Lloyd et al., 2006; Neel, 1962), presumably developed as a result of natural selection driven by the ‘feast and famine’ ecology of its Ossabaw Island habitat with a high seasonal variation in food supply (Brisbin and Mayer, 2001).

Humans are speculated to have a ‘thrifty’, obesity-promoting genotype like that seen in the Ossabaw pig shaped by a similar type of feast and famine environment during the hunter-gatherer stage of human development (Neel, 1962) and being challenged by the food surplus of the westernized lifestyle of the last 50–60 years (Bluher, 2019). Thus, the indication that the Ossabaw pig harbors a thrifty genome further adds to the relevance of this pig for modeling human obesity. It is therefore of utmost interest to map the Ossabaw pig genome to understand the genomic underpinnings of the thrifty/obese phenotype. However, until now, specific information on the Ossabaw pig genome has been limited. A genetic link to the Canary Island Black pig breed and, notably, a predominant impaired function mutation (V199I) in the *PRKAG3* gene coding for the gamma subunit of AMPK (AMP-activated protein kinase) were published by Lloyd et al. (2006). This AMPK genotype was previously described to be associated with increased intramuscular fat and low muscle glycogen (Andersson, 2003; Ciobanu et al., 2001). It is worth noting that the minority of Ossabaw pigs not being homozygous for this mutation still get obese and most show MetS signs (Chawla et al., 2012). On the other hand, it is estimated that 30% of obese V199I homozygous mutant Ossabaw pigs do not develop insulin resistance (Sham et al., 2014). This is consistent with epidemiological data on obese humans (Adult Obesity Facts, 2021) and illustrates the complex genetics of obesity and obesity associated disease, and searching for other mechanistic clues elsewhere in the Ossabaw pig genome is therefore highly relevant. Here we report the whole genome sequencing of the Ossabaw pig and identify possible mutations and candidate genes that may be related to obesity, providing a valuable resource for future research.

## Results

### *De novo* genome assembly and annotation

**Figure 1:**
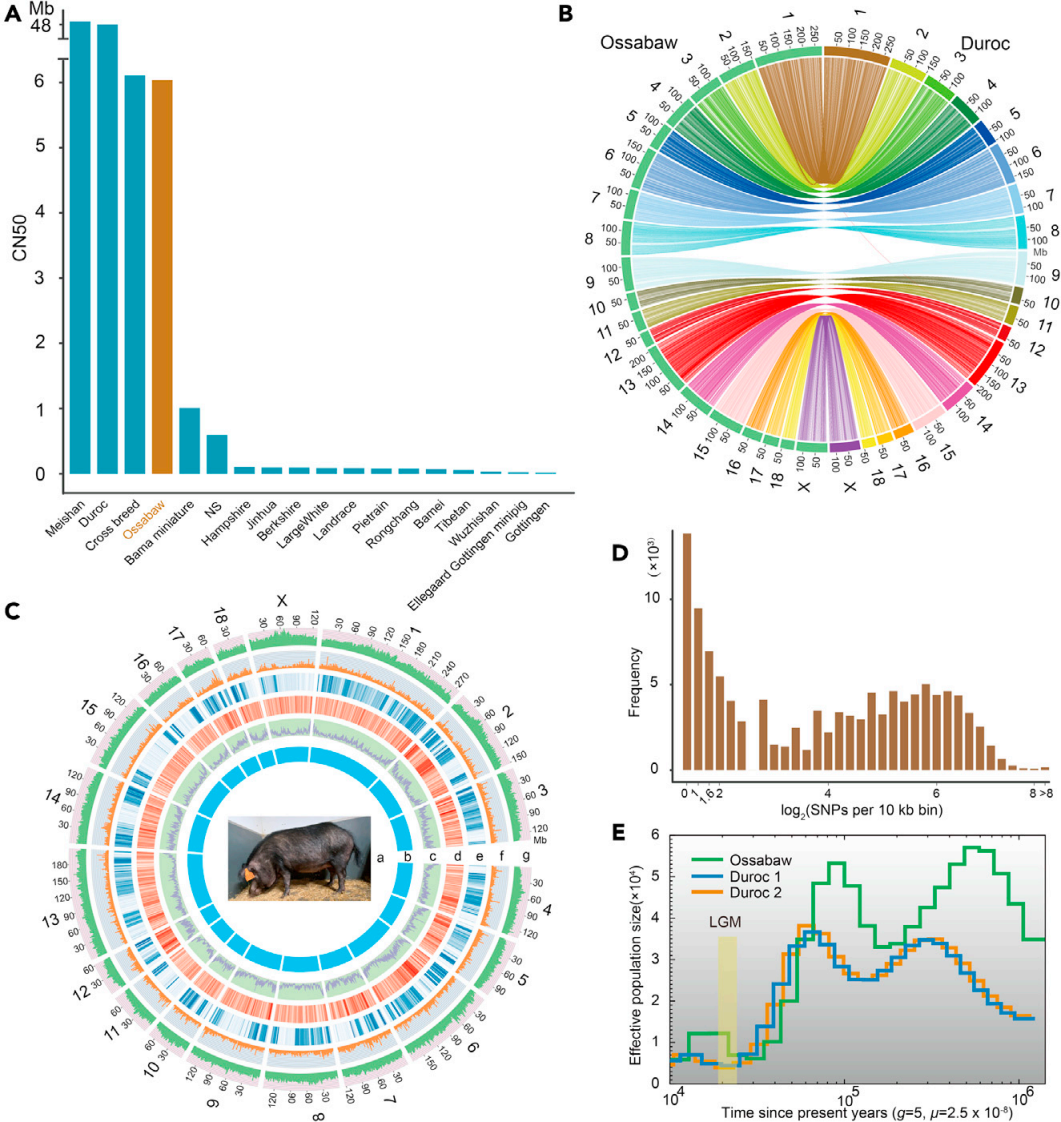
Genome assembly landscape of Ossabaw pig genome (A) Contig N50 of Ossabaw pig and 17 published genomes of other pig breeds. The contig N50 of Ossabaw is much longer than most of (14 out of 17) published pig genomes. This result suggests our sequencing to be effective and economic. (B) Circos plot of Ossabaw pig and Duroc chromosomes. (C) Distribution of genome elements of Ossabaw pig. a, photo of the Ossabaw pig 2702 (Donna). b, 19 chromosomes. c, GC content at 100 kb bins. d, gene density at 500 kb bins. e, SNP density at 500 kb bins. f, histogram of DNA transposon ratios. g, histogram of retrotransposon ratios. (D) Frequency distribution of SNP per 10 kb bin. (E) Estimated effective population size (Ne) of Ossabaw pig using heterozygous SNPs. LGM: Last Glacial Maximum.

To ensure a high quality whole Ossabaw pig genome, single-tube long fragment read (stLFR) (Wang et al., 2019) and Oxford Nanopore Technology (ONT) sequencing strategies were combined based on long DNA fragments (main band: ∼100 kb) (Figure S1) prepared from peripheral blood mononuclear cells from one individual female Ossabaw pig housed at the DTU Ossabaw facility, Risø, Denmark. The stLFR and ONT libraries were sequenced on BGISEQ-500 and GridION X5 platforms, respectively. In total, we generated 305.83 Gb paired-end short reads (read1: 100bp; read2: 142 bp) and 24.18 Gb ONT long reads with an N50 of 33.5 Kb (Table S1). The initial assembly was carried out using the Supernova algorithm (Weisenfeld et al., 2017) on short paired-end reads, generating a draft genome (V1) with total length, contig N50 size (CN50) and scaffold N50 size (SN50) of 2509.23 Mb, 119.35 Kb and 21.82 Mb, respectively. To exclude any redundant assembly caused by heterozygosity, we tidied the initial assembly using Redundans (Pryszcz and Gabaldón, 2016), removing ∼58 Mb sequences (V2). Then we filled assembled gaps by using stLFR_GapCloser based on short paired-end reads, enhancing CN50 to 175.00 Kb (V3). To take advantage of ONT reads, we firstly conducted error-base correction by using 130 Gb short reads with two rounds of Pilon (Walker et al., 2014) running. Next, the corrected long reads were mapped to the V3 genome to fill gaps using TGS-GapCloser (Xu et al., 2019). This effort increased the quality of the assembled genome to a CN50 of 6028.60 Kb and an SN50 of 22.31 Mb (V4) (Table S2), which is considerably higher than most published pig genomes (14 out of 17) (Figure 1A and Table S3). Finally, RaGOO (Alonge et al., 2019) was used to anchor assembled scaffolds to chromosomes based on the Duroc genome (*Sus scrofa* reference genome, NCBI accession: GCA_000003025.6, Sus scrofa 11.1), generating 19 chromosomes ranging from 56.78 Mb to 277.92 Mb (Table S4, Figure 1C) and 313 unplaced scaffolds with a total length of 31.76 Mb, which means that ∼98.7% of scaffolds were anchored. The final assembly (V5) contains 2450.37 Mb bases with gaps of 36.28 Mb and SN50 of 140.68 Mb (Table S3). The quality of the genome assembly can be summarized in the following seven parameters: (1) ∼98% of the sequenced short paired-end reads can be mapped back to the assembled genome indicating that most of the repetitive sequences were assembled. (2) Evaluation of base accuracy by calling homozygous SNPs and INDEls indicates the base inaccuracy to be ∼0.0002% (Table S4), well below the standard of 0.01% for human genome (Schmutz et al., 2004). (3) The single peak distribution of sequencing depth as a function of GC content (Figure S2) suggests that this assembly has no contamination and no redundancy. (4) GC content distribution is similar to that of the Duroc genome (Figure S3). (5) The assembled genome size is very close to the size of the Duroc genome (∼2458.36 Mb) (excluding chromosome Y) (Table S3) and also chromosome lengths are very similar between the Ossabaw and the Duroc genome (Table S5). (6) The Ossabaw genome has a good coverage and syntenic relationship with Duroc (Figure 1B). (7) ∼96% of Benchmarking Universal Single-Copy Orthologs (BUSCO) (Simao et al., 2015) can be detected in the assembly suggesting that most of the genes were assembled (Table S6). All of this underlines the high quality of the Ossabaw genome assembly.

Next, the genome was annotated (including repeat detection and gene prediction) using both *de novo* prediction and homology-based methods (see details in STAR Methods section). The result shows that ∼38.22% of the genome is annotated as transposable elements (TEs) composed by DNA Transposon/Long Interspersed Nuclear Element/Short Interspersed Nuclear Element/Long Terminal Repeat of ∼0.98%, ∼36.05%, ∼0.21% and ∼2.69%, respectively (Table S7). The sequence divergence distribution of predicted TEs compared to the Repbase database (Jurka et al., 2005) is shown in Figure S4. After masking repeat sequences, we finally predicted 21,794 protein coding genes. The mean mRNA, coding sequence, exon and intron lengths distribution were similar to other mammalian genomes as well as the Duroc genome (Figure S5). Short non-coding RNAs were also identified and shown in Table S8. Moreover, a BUSCO completeness score of 98.5% and a duplicated score of only 0.5% (Table S9) of the annotated Ossabaw genes indicate that the gene annotation is of high quality, allowing further evolutionary analysis to be done with high certainty. Finally, we mapped protein sequences of the predicted genes to KEGG (Kanehisa and Goto, 2000), Swiss-Prot (Boeckmann et al., 2003), InterPro (Apweiler et al., 2001), and TrEMBL (Boeckmann et al., 2003) databases to assign related functions to these genes, generating a function annotation percentage of ∼98.11% (Table S10).

### Analysis of SNPs and SNP based effective population size during evolution

The Genome analysis toolkit (GATK) (McKenna et al., 2010) pipeline was used to call single nucleotide polymorphisms (SNPs) in the Ossabaw (V5) genome and 4,039,107 heterozygous SNPs were identified corresponding to a nucleotide diversity of ∼1.68‰, which is ∼2.4 times higher than the human genome (0.69‰) (Venter et al., 2001) but lower than the Göttingen minipig whole genome SNP rate (2.44‰) (Vamathevan et al., 2013). The SNPs density and distribution are shown in Figures 1C and 1D. Based on the identified SNPs, we estimated changes in the effective population size (Ne) using the pairwise sequentially Markovian coalescent (PSMC) model, a method which can infer population sizes from approximately 10,000 to 1 million years ago (MYA) (Li and Durbin, 2011). We found two peaks with Ne of ∼5.7 × 10^4^ and ∼5.3 × 10^4^ at ∼600 × 10^3^ and 90 × 10^3^ years ago (Figure 1E) which is ∼2 times higher than other wild boars in Eurasia (Groenen et al., 2012). However, after the most recent peak, the Ne decreased rapidly to ∼0.6 × 10^3^–30 × 10^3^ years ago. Then the Ne increased to ∼1.3 × 10^4^ although this species had experienced the Last Glacial Maximum (LGM; 20,000 years ago), (Yokoyama et al., 2000) (Figure 1E) which is also different from other pig breeds; however the Ne decreases again at ∼4000 years ago. To validate the analysis, we downloaded sequencing data of two Duroc individuals (Table S11), executed PSMC estimation in the same way as was done for the Ossabaw genome and obtained Ne results (Figure 1E) similar to those obtained from other genomes (Groenen et al., 2012).

Then we further investigated the distribution of SNPs by combining with gene annotation results. We found that ∼36% (1,464,991, Table S12) of the SNPs were located in gene regions, of which 33,079 were in exon regions. Of these 33,079 SNPs, 24,617 were Ossabaw pig specific and 10,688 caused changes in amino acids in the protein products of 3,781 genes, potentially affecting their function. Then we analyzed the function of these genes by carrying out KEGG enrichment analysis and found 1,811 genes to be enriched in 335 pathways (Figure S6; Table S13). The top 20 (based on *p-*value) pathways include “Olfactory transduction (347 including 342 olfactory receptor (OR) genes)”, drug metabolism related “Metabolism of xenobiotics by cytochrome P450 (21 genes)”, immune system related “Cell adhesion molecules (CAMs) (53 genes),” and “Antigen processing and presentation (28 genes)”. Olfaction is a successful product of evolution and a complex process. Pigs have excellent olfactory abilities as they have ∼1,100 functional OR genes (Nguyen et al., 2012) and olfaction could help them forage, seek mate, and evade hazardous substances or predators. The large number of heterozygous loci increases the variety of OR genes of Ossabaw pigs, which may benefit survival of their population. Xenobiotics metabolizing enzymes such as *CYP1A2* (Azab et al., 2018), *UGT2A1* (Azab et al., 2018), *UGT1A6* (Ciotti et al., 1997), *ALDH3A1* (Glatt et al., 2008), *ALDH3B1* (Marchitti et al., 2010), and *EPHX1* (Yang et al., 2014) are important for detoxification, and in the Ossabaw genome they all contain heterozygous loci. The heterozygosity of major histocompatibility complex (MHC) related genes including *SLA-2*, *SLA-8*, *SLA-6*, *SLA-DRA*, *SLA-DQA1*, *SLA-DQB1*, *SLA-DRB1*, *SLA-DOB* and *TAP2*, involved in recognition, processing and presentation of antigens may contribute to the flexibility and robustness of the immune system in the Ossabaw pig. Advantages of heterozygous genes have been described in many mammals such as mice (Han et al., 2008), cattle, sheep, horse, and dog (Hedrick, 2015); therefore, the heterozygosity of these pivotal genes may help Ossabaw pig to maintain population size in challenging environments such as their natural habitat.

Obesity is a conspicuous trait of the Ossabaw pig and heterozygosity of *POMC*, *LEP* and *LEPR* genes were reported to impact the obesity of humans and mice (Chung et al., 1998; Farooqi et al., 2006). We therefore examined the heterozygosity of related genes associated with lipid metabolism and energy homeostasis in the Ossabaw genome and found that 56 heterozygous genes (Table S14) were associated with fatty acid metabolism. For example, *CPT1A*, encoding carnitine palmitoyltransferase 1A, is essential for long-chain fatty acid oxidation and deficiency may result in fatty acid oxidation disorders and accumulation of fatty acids (Bennett and Santani, 2016). *HACD1*(3-hydroxyacyl-CoA dehydratase 1) encodes an enzyme involved in very long-chain fatty acid elongation (Ikeda et al., 2008). *ADIPOQ* (adiponectin, C1Q and collagen domain containing), which is expressed in adipose tissue exclusively, is centrally involved in the control of adipose metabolism and its dysregulation may lead to type 2 diabetes and non-alcoholic fatty liver disease (NAFLD) (Renaldi et al., 2009; Ukkola and Santaniemi, 2002). *ACSBG2* (Long-chain-fatty-acid CoA ligase) catalyzes the conversion of fatty acids such as (very-) long chain fatty acids to acyl-CoAs for both synthesis of cellular lipids, and degradation via beta-oxidation (Mashek et al., 2007). These heterozygous key genes in lipid metabolism potentially play a role for the “thrifty” phenotype of the Ossabaw pig and should be further investigated.

### Gene family analysis among representative mammals

**Figure 2:**
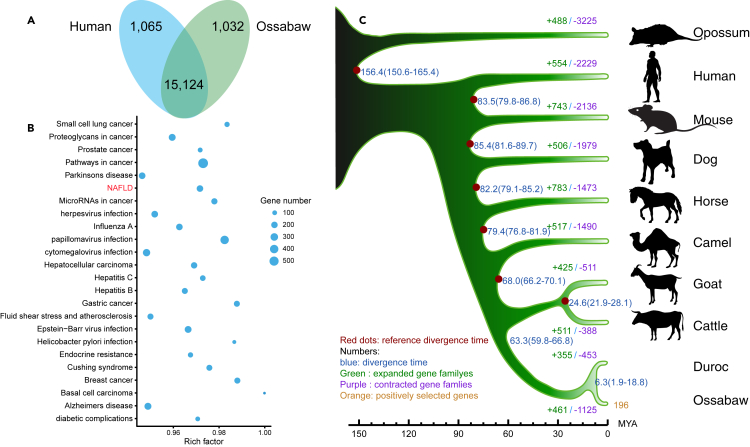
Gene evolution of the Ossabaw pig and representative mammals (A) Gene families of Ossabaw pig and human. (B) Genes related to human disease of Ossabaw pig genome. All enrichment *q-*values are below 0.05. (C) Phylogenetic tree, divergence time, gene families of the Ossabaw pig and representative mammals. Red dots: seven reference time points from TimeTree database (Hedges et al., 2006).

Pigs are considered good animal models of complex human diseases and for the definition of disease associated genotypes, and it is therefore of interest to compare the Ossabaw genome to the human genome. Enrichment analysis (Beissbarth and Speed, 2004; Chen et al., 2010) of the genes shared between the two species (Figure 2A) revealed that 1,799 genes were significantly enriched in Human Disease pathways (*q-*value < 0.05) (Figure 2B), suggesting the potential of Ossabaw pigs to become models of the diseases related to these genes. Specifically, the NAFLD pathway contains 208 Ossabaw genes (Table S15). Atherosclerosis genes were also enriched in human disease pathways (Figure 2B). Human non-alcoholic steatohepatitis (NASH) related MetS and abnormal liver histology and atherosclerosis have been experimentally induced by a diet with high calories, fat, fructose, and cholesterol in Ossabaw pigs (Lee et al., 2009). These findings make the regulation of these genes during disease development highly interesting, also with respect to identifying biomarkers for disease, especially early disease stages. Notably, almost one-third of the enriched pathways include host response to viral infections comprising both DNA viruses as herpes and hepatitis virus and the influenza A RNA virus.

In addition to human, we also compared Ossabaw gene families with another eight representative mammals including mouse, dog, horse, camel, cattle, goat, Duroc (pig) and opossum as an outgroup (Table S16). We identified 18,282 gene families across all 10 species, including 10,418 single copy orthologous genes (SCOGs) (Figure S7). For the Ossabaw pig, 461 gene families expanded, whereas, 1,125 gene families contracted from their most recent common ancestor (MRCA) (Figure 2C). By using the 10,418 SCOGs, we constructed a phylogenetic tree for ten species employing a Bayesian method (bootstrap values of all clades are 100) (Figure 2C). Unsurprisingly, this analysis placed Duroc and Ossabaw pig breeds in the same branch separately from other species. Then we estimated the divergence time of Duroc and Ossabaw to be ∼6.3 MYA using 7 reference time points from the TimeTree database (Hedges et al., 2006) for calibration. For the SCOGs, we further investigated the evolutionary pressure acting on them and identified 196 positively selected genes (PSGs) (Table S17) in the Ossabaw pig lineage. These genes were significantly (*q-*value < 0.05) enriched in GO (Gene Ontology) terms cellular component (GO:0005575, 41 genes, including intracellular part, DNA repair complex and membrane-bound organelle), and primary metabolic processes (GO:0044238, 54 genes such as tricarboxylic acid cycle, citrate metabolism and DNA metabolism). For example, *CRTC3* (CREB-regulated transcription coactivator 3), positively regulates CREB dependent gene transcription and has been reported to link to weight gain and adiposity of humans (Song et al., 2010). *PMS2* (mismatch repair endonuclease PMS2), functions as a protective mediator of cell survival and modulates protective DNA damage response (Fedier et al., 2002). *VAPB* (vesicle-associated membrane protein-associated protein B) is involved in the IRE1/XBP1 signaling of the unfolded protein response because of ER stress and suppressing accumulation of unfolded/misfolded proteins (Kanekura et al., 2006). *XRCC6* (X-ray repair cross-complementing protein 6), encoding Ku70 dimerized with Ku80, is required for the non-homologous end-joining of DNA repair, V(D)J recombination of mammalian immune system and telomere length maintenance (Boulton and Jackson, 1998) and plays an important role in longevity assurance (Bernstein et al., 2008). *PTGS2* (prostaglandin G/H synthase 2 precursor), expressed during inflammation and tumorigenesis, is associated with increased cell adhesion, negatively regulating intrinsic apoptosis in response to osmotic stress and resistance to tumor angiogenesis. Such positively selected sites of *CRTC3* and stress response genes, *PMS2*, *VAPB*, *XRCC6* and *PTGS2* are examples of genes that may improve the ability of adapting to environmental stressors, such as periodic scarcity of food, high salt diets, and minimal availability of freshwater, all of which have been pivotal to the survival of the Ossabaw pig on Ossabaw Island. The positively selected PRKAG3 gene encodes the gamma subunit of AMPK and is intriguing as its impaired function mutation (V199I) is associated with increased adiposity and low muscle glycogen (Andersson, 2003; Ciobanu et al., 2001), potentially favoring adaptation to periodic food scarcity. No feral Ossabaw pigs sampled on Ossabaw Island have been found to be homozygous for the wild type gene (V199) (Lloyd et al., 2006).

### Genomic variations compared to Duroc genome

Based on our high quality Ossabaw genome assembly, we further identified genomic variations including single nucleotide polymorphisms (SNPs), insertions, deletions and inversions in Ossabaw pig genome compared to the Duroc reference genome (Groenen et al., 2012), providing a valuable resource for further study.

**Figure 3:**
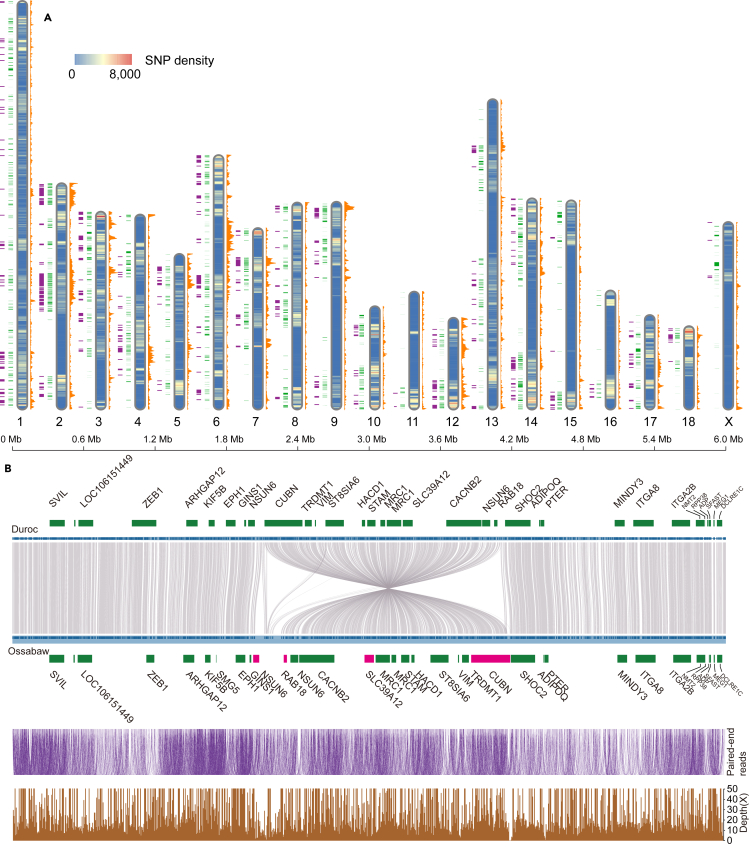
Genomic variations of Ossabaw pig compared to Duroc pig genome (A) SNPs, genes, and higher density regions along the Duroc genome. Each chromosome contains four datasets. From left to right: Purple, higher density regions (*see details in main text*). Green, genes with nonsynonymous SNPs. Chromosome bar, distribution of SNP density at 500 kb bins. Orange, gene density at 500 kb bins. (B) A ∼2 Mb inversion on chromosome 10 between Ossabaw and Duroc genomes. Green rectangles, genes in the ∼6 Mb region comprising the inversion. Pink rectangles, pseudogenes. Purple curves, mapping relationship of paired-end reads. Brown, sequencing depth of ONT reads mapped to this region (also see Figure S9).

We mapped ∼52-fold sequencing reads of the Ossabaw pig genome to the Duroc genome using the GATK pipeline (McKenna et al., 2010) and identified a total of 3,987,393 SNPs including 2,579,363, 1,382,141 and 27,484 in intergenic, intron and exon regions, respectively (Table S18). The distribution of SNPs and gene density is shown in Figure 3A at 500 kb bins. We found 536 bins (268 Mb of genome, Table S19) to contain a high density of SNPs (∼1977.4 SNPs per bin as compared to the overall average of ∼794.8) and genes (∼10.3 genes per bin; average is ∼4.0). There are eight regions larger than 5 Mb which consist of consecutive bins (Figures 3A and Table S20). The high SNP density in these regions indicates that the genes contained therein might have evolved fast because of higher mutation rate of these regions. We therefore explored gene functions of the 5,271 genes (24.19% of whole genome genes) located in these regions by enrichment analysis. This revealed that 3,930 genes could be mapped to 3,469 GO terms (74 terms enriched significantly, i.e. with *q-*value < 0.05) (Table S21). The top 30 GO terms are shown in Figure S8, illustrating that 656 genes were enriched in receptor activity (GO:0004872) including 490 genes involved in olfactory transduction. In addition, 853 and 81 genes were enriched in cellular responses to stimulus (GO:0050896) and immune responses (GO:0006955) respectively, highlighting the potential adaptability of the Ossabaw pig phenotype to changing environmental conditions.

Then we mapped structure variations (length >50 bp) in the Ossabaw pig genome using smartie-sv (Kronenberg et al., 2018), finding 13,875 insertions and 21,700 deletions, of which 4,899 and 7,637, respectively were located in gene regions (Figure S9). Moreover, 25 insertions and 289 deletions were found to break exons in 250 genes and 2 genes contained both insertions and deletions. It should be noted that 58 of the 250 genes are associated with obesity or diabetes (Table S22). For example, *NLRP3* (NACHT, LRR and PYD domains-containing protein 3), has a deletion of 20,668 bases, leading to a truncation of this gene. *NLRP3* has been reported to have a role in protecting against high fat diet-induced obesity and insulin resistance (Rheinheimer et al., 2017). *APOL3* (apolipoprotein L3) has a 3,236 bp deletion including the third exon. This gene affects the transport of lipids (Hornbeck et al., 2012). Thus, indel mutations of these genes may affect obesity related functions and contribute to the obesity susceptibility of Ossabaw pigs and have potential as biomarkers of metabolic diseases.

As an exception to the overall high synteny between the Ossabaw and Duroc genomes (Figure 1C), we found a ∼2 Mb inversion in the Ossabaw genome located on chromosome 10 (Figures 3B and S10) as identified both by paired-end short reads and ONT sequencing long reads. Comparing the Ossabaw genome to the other 14 pig breeds (Figure S11) proved the reliability of this inversion. We found this inversion to be conserved in all individual Ossabaw genomes (N = 49) recently investigated by us (Y.Z. et al., unpublished data). This inversed ∼2 Mb region includes 13 genes and the inversion may be associated with the pseudogenization of four genes (Table S23), including the *CUBN* (Cubilin) gene which was interrupted. This gene is associated with albuminuria in patients with diabetes (Böger et al., 2011) and plays a potential role in type 2 diabetes susceptibility (Tsekmekidou et al., 2020). In addition, included is *RAB18* (Ras-related protein Rab-18), acting as a molecular switch between lipogenesis and lipolysis, and having a central role in controlling storage and release of fat and thereby in the development of obesity (Pulido et al., 2011). *SLC39A2* and *NSUN6*, participating in metal ion transportation and RNA transmethylation, also should be noticed in future research. In addition, figuring in genes within ∼2 Mb flanking region on each side of the inversion, more than one-third of total 34 genes located here (Figure 3B) are obesity or diabetes related, including *VIM*, *PTER*, *ZEB1*, *ITGA2B*, *KIF5B*, *MRC1*, *CACNB2*, *NMT2*, *HACD1* (different locus from the one mentioned above), *SVIL* and *TRDMT1*. Gene interaction is a well-known phenomenon of closely located genes (Mani et al., 2008) conferring significance to these observations of obesity and diabetes related (pseudo-) genes affected by the Ossabaw specific gene inversion, implying them in the “thrifty” phenotype of the Ossabaw pig.

### Evolution of the Ossabaw genome compared to other pig breeds

**Figure 4:**
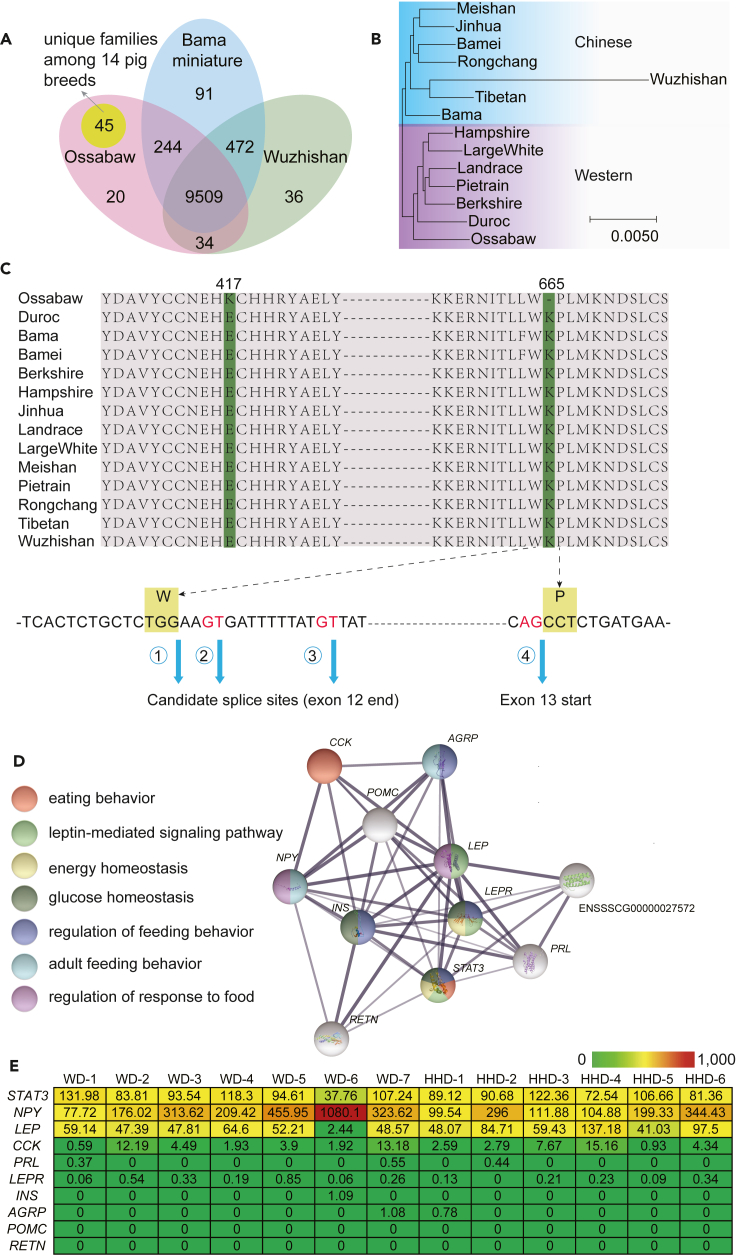
Gene evolution among 14 pig breeds (A) gene families among three miniature pig breeds. (B) phylogenetic tree of 14 pig breeds (also see Figure S12). (C) positively selected site of Ossabaw *LEPR* gene. ①, ②, ③ represent possible splice sites of exon 12 (also see Figures S13 and S14). (D) gene network of *LEPR*. This network was referenced from the STRING database (https://string-db.org/). The colored circles represent the corresponding biological processes to which these genes are related, and the thickness of lines represents the strength of gene interaction. (E) Heatmap of gene expression (FPKM, Fragments Per Kilobase of exon model per Million mapped fragments) for *LEPR* network genes, from an Ossabaw diet experiment (Walker et al., 2019), WD (Western style diet) and HHD (Heart Healthy style diet) group.

To further substantiate our findings, we moved forward to compare the Ossabaw genome with genomes of another 13 pig breeds with BUSCO completeness higher than 90% (Figure S12). We also defined gene families within 14 pig breeds (including Duroc and Ossabaw), generating 10,532 gene families of which 5,113 were SCOGs (single copy orthologous genes, Figure S13). The three mini pig breeds Ossabaw, Bama miniature and Wuzhishan shared 9,509 gene families and Ossabaw pig had 65 unique ones (Figure 4A), of which 45 were unique among 14 pig breeds (Table S24). When analyzing the gene function of gene families unique for Ossabaw pigs, we found two important families (eight copies of epithelial chloride channel protein-like and 36 copies of cyclic nucleotide-gated cation channel beta-3 (*CNGB3*)) to be involved with ion regulation. Chloride channels involved in the renin secretion pathway, are vital structures for sustaining ion homeostasis, cell volume, transepithelial transport and regulation of electrical excitability (Jentsch et al., 2002). *CNGB3* belongs to one of the beta subunits of cyclic nucleotide–gated ion channels (CNG) which have been discovered in various tissues and mainly function in sensory transduction (Kaupp and Seifert, 2002), and may also contribute to maintain homeostasis helping the Ossabaw pig to cope with the high salt concentration of the drinking water of its habitat (Stribling et al., 1984). We also found *CYCS* (cytochrome *c*) gene expanded with 14 copies in the Ossabaw pig while other pig breeds have less than 9 copies (Figure S14). *CYCS* is important for regulated cell death (apoptosis) (Tang et al., 2019).

We constructed phylogenetic trees for the 14 pig breeds using sequences of 5113 SCOGs with different models (GTR, KHY and JC) and three of four results generate consistent trees (Figures 4B and S15). We identified 214 PSGs in the Ossabaw pig genome compared to the other pig genomes. Among the PSGs, we found the *LEPR* (leptin receptor) gene which is known to be a key gene associated with obesity and diabetes (Chua et al., 1996; Li et al., 2017; Pereira et al., 2019; Zhang et al., 2018). The Ossabaw *LEPR* gene contained two positively selected sites (Figure 4C) which were strongly supported by sequencing reads (Figures S16 and S17). The first of these sites led to a change of the amino acid E (glutamic acid, acidic) to K (lysine, alkaline) (E417K) which is a very non-conservative amino acid substitution. The second mutation may lead to three types of translation according to the GT-AG splice rule (Breathnach et al., 1978; Breathnach and Chambon, 1981). The first splice site GG, violating the GT-AG rule, will generate a deletion of one amino acid after W664. The second and third possible sites will shift frames, making the gene into a pseudogene. Published RNA sequencing data from an Ossabaw diet experiment showed that *LEPR* gene was minimally expressed in 13 examined Ossabaw pigs (epicardial adipose tissue) (Walker et al., 2019) (Figure 4E). Also, six genes (*CCK*, *PRL*, *INS*, *AGRP*, *POMC* and *RETN*) were found in the same study not to be expressed. As indicated in Figure 4D these genes are all associated with *LEPR* so even if *LEP* was found to be expressed both in WD (Western style diet) and HHD (Heart Healthy style diet) groups (Figure 4E), the silence of related upstream regulatory or downstream receptor genes is highly likely to affect the function of pathways related to *LEPR*. More samples from different tissues should be checked in future work. The importance of leptin genes and modulation of leptin action and protein expression in Ossabaw pigs were indicated in previous studies (Lee et al., 2009; McKenney et al., 2014; Neeb et al., 2010; Payne et al., 2010), providing stimulus for future obesity and disease studies on these genes.

## Discussion

In the present study, we selected an economical sequencing strategy, combining sequencing of a stLFR library giving ∼125-fold coverage of the genome with “low” depth (around 10-fold) ONT data instead of a more expensive high depth ONT or Pacbio sequencing. The fully *de novo* assembled Ossabaw pig genome (V4) reached a CN50 of ∼6 Mb which rivals assembling using high depth ONT or Pacbio sequencing. Except for the Duroc reference genome (Warr et al., 2020) and two other published pig genomes (Figure 1A), the Ossabaw genome assembly presented here greatly surpasses all other published pig genomes with respect to contig length, including those of miniature pigs specifically bred for biomedical purposes. Thus, the genome of the highly inbred Chinese Bama miniature pig has been assembled at the chromosome level with a CN50 of 1 Mb (Zhang et al., 2019), whereas the Wuzhishan miniature pig (Fang et al., 2012) has only been reported at the scaffold level with a CN50 of 0.024 Mb. Finally, the genome of the European Göttingen minipig, widely used for preclinical research and toxicology has been assembled at CN50 of 0.021 Mb (Vamathevan et al., 2013) This high quality Ossabaw pig genome assembly allows further analyses to be done with high certainty.

The overall SNP rate of the Ossabaw pig genome (∼1.68‰) is higher than that of the human genome (0.69‰), and Chinese miniature pig breeds Bama (0.01‰), and Wuzhishan (1.4‰) (Fang et al., 2012; Zhang et al., 2019), but considerably lower than the Göttingen minipig genome (2.44‰) and also a little lower than the reference Duroc pig genome (1.75‰). The high SNP rate of the Ossabaw genome results in the heterozygosis of important genes, such as fatty acid metabolism related genes including *CPT1A*, *HACD1*, *ADIPOQ* and *ACSBG2*. Just as in humans and mice, these heterozygous genes are involved in fatty acid metabolism and thus may contribute to the Ossabaw obese phenotype. These interesting heterozygous genes potentially play a role in the ‘thrifty’ phenotype of the Ossabaw pig and should be further investigated.

When comparing genes in the Ossabaw genome to the other 13 pig breeds, 214 genes were identified as positively selected. One of these is the *LEPR* gene encoding the leptin receptor, centrally involved in many aspects of appetite control, lipid turnover and innate immune reactions in adipose tissues. Two sites in the *LEPR* gene are positively selected; the first one gives rise to a change in from glutamic acid to lysine at amino acid 417 (E417K). The second gives rise to three possible alternative translation products, one resulting in a deletion of AA665, and the two other ones frameshifting the read, abolishing translation (pseudogenization). Analysis of existing RNA data showed that *LEPR* was minimally expressed in examined samples (Walker et al., 2019). However, more samples from different tissues should be checked and functional experiments should be done in the future to investigate the consequence of these changes in the Ossabaw *LEPR* gene.

There is good evidence that translation of obese Ossabaw pig biology to human medicine is substantial (Neeb et al., 2010; Sham et al., 2014; Sturek et al., 2015, 2020). Indeed, its propensity for obesity has likely been shaped by the feast-and-famine environment of Ossabaw Island. A major food source of the Ossabaw pig is the acorn, which has seasonal variation (Mayer and Brisbin, 1991). The pig is monogastric, thus cannot derive sufficient calories from consumption of grass and other cellulose-based foods that require fermentative digestion. Ossabaw Island mimics the evolutionary forces shaping the human genome during the hunter-gatherer stage of human evolution (Neel, 1962). This makes the Ossabaw pig genome highly interesting in the search for molecular clues to predisposition to obesity and its comorbidities, including the obesity-associated increased risk of adverse outcomes during viral respiratory disease such as influenza and COVID-19 (Heegaard et al., 2020; Popkin et al., 2020). The high quality Ossabaw pig genome presented here adds a highly human relevant obesity prone genome to the porcine genetic engineering toolbox increasingly used for diabetes and metabolic research (Zettler et al., 2020). This allows new, relevant gene targets to be defined and provides precise information for designing genetic modifications for probing gene functions. As an example, genes related to innate immune settings during immune training can now be analyzed for expression patterns and functionality during obesity and comorbidity development (Bekkering et al., 2020) in the Ossabaw pig and other pig breeds.

The Ossabaw pig genome provides a blueprint to inform gene modification and RNA targeting/silencing experiments, targeted mRNA quantification and ultimately, by inference, investigations of the Ossabaw proteome in health and disease. The availability of the genome of the obesity-prone Ossabaw miniature pig has the potential to greatly promote our understanding of complex immunological and metabolic features, and comorbidities in obesity.

### Limitations of the study

Functional studies should be performed to validate the candidate genes that were found in this study to be associated with the tendency of the Ossabaw miniature pig to obesity, to further consolidate its use as an outstanding animal model for human obesity and obesity related disease. As with any animal model, interspecies differences should be noted when using the model to investigate mechanisms of human diseases.

## STAR★Methods

### Key resources table

**Table:** 

REAGENT or RESOURCE	SOURCE	IDENTIFIER
**Biological samples**		

Ossabaw pig	This study	NA

**Deposited data**		

Sequencing data for Ossabaw pig	This study	CNGB Sequence Archive (https://db.cngb.org/cnsa/) under the accession number CNP0001681
Genome assembly for Ossabaw pig	This study	CNGB Sequence Archive (https://db.cngb.org/cnsa/) under the accession number CNA0020809

**Software and algorithms**		

SOAPfilter v2.2	(Luo et al., 2012)	https://github.com/aquaskyline/SOAPdenovo2
Supernova v2.11	(Weisenfeld et al., 2017)	https://support.10xgenomics.com/de-novo-assembly/software/downloads/latest
Redundans	(Pryszcz and Gabaldón, 2016)	https://github.com/lpryszcz/redundans
stLFR_GapCloser	BGI-Qingdao	https://github.com/BGI-Qingdao/stLFR_GapCloser
Pilon v1.2.3	(Walker et al., 2014)	https://github.com/broadinstitute/pilon/releases/
TGS-GapCloser	(Xu et al., 2019)	https://github.com/BGI-Qingdao/TGS-GapCloser
RaGOO v1.1	(Alonge et al., 2019)	https://github.com/malonge/RaGOO
RepeatMasker v4.0.5	(Tarailo-Graovac and Chen, 2009)	http://www.repeatmasker.org/
RepeatModeler v1.0.8	(Tarailo-Graovac and Chen, 2009)	http://www.repeatmasker.org/RepeatModeler/
LTR-FINDER v1.0.6	(Xu and Wang, 2007)	https://github.com/oushujun/LTR_FINDER_parallel
Tandem Repeat Finder v4.0.7	(Benson, 1999)	https://tandem.bu.edu/trf/trf.html
BLAT v36	(Kent, 2002)	https://genome.ucsc.edu/cgi-bin/hgBlat?command=start
GeneWise v2.4.1	(Birney et al., 2004)	https://www.ebi.ac.uk/∼birney/wise2/
Augustus v3.1	(Keller et al., 2011)	http://augustus.gobics.de/
EVidenceModeler v1.1.1	(Haas et al., 2008)	https://evidencemodeler.github.io/
BUSCO v2.0	(Simao et al., 2015)	https://busco-archive.ezlab.org/v2/
BLAST v2.2.26	(Altschul et al., 1990)	https://blast.ncbi.nlm.nih.gov/Blast.cgi
BWA-MEM v0.7.12	(Li and Durbin, 2010)	https://github.com/lh3/bwa
SAMtools v0.1.19	(Guindon et al., 2009)	https://github.com/samtools/samtools
Picard package v1.54	Broad Institute	https://broadinstitute.github.io/picard/
GATK v4.1.2	(McKenna et al., 2010)	https://gatk.broadinstitute.org/hc/en-us
PSMC v0.6.5	(Li and Durbin, 2011)	https://github.com/lh3/psmc
TreeFam v1.1	(Li et al., 2006)	http://www.treefam.org/
CAFÉ v2.1	(De Bie et al., 2006)	https://github.com/hahnlab/CAFE
PAML v4.9	(Yang, 2007)	http://abacus.gene.ucl.ac.uk/software/paml.html
MUSCLE v3.8.31	(Edgar, 2004)	https://www.ebi.ac.uk/Tools/msa/muscle/
PRANK v100802.	(Löytynoja, 2014)	https://www.ebi.ac.uk/research/goldman/software/prank
gBlocks v0.91b	(Talavera and Castresana, 2007)	https://github.com/atmaivancevic/Gblocks
LastZ v1.1	(Harris, 2007)	http://www.bx.psu.edu/miller_lab/dist/README.lastz-1.02.00/README.lastz-1.02.00a.html
Smartie-sv	(Kronenberg et al., 2018)	https://github.com/zeeev/smartie-sv
SOAPnuke v1.6.5	(Chen et al., 2017)	https://github.com/BGI-flexlab/SOAPnuke
Bowtie2	(Langmead and Salzberg, 2012)	https://github.com/BenLangmead/bowtie2
RSEM v1.2.31	(Li and Dewey, 2011)	https://github.com/deweylab/RSEM

### Resource availability

#### Lead contact

Further information and requests for materials should be directed to and will be fulfilled by the lead contact, Peter M.H. Heegaard (pmhh@dtu.dk).

#### Materials availability

This study did not generate new unique reagents.

### Experimental model and subject details

#### Source organism: *Sus scrofa*

Genomic DNA was isolated from an EDTA stabilized blood sample of a female Ossabaw pig. The sample was collected according to Danish legislation and under the Danish Animal Experiments Inspectorate approval 2016-15-0201-01022. The isolated genomic DNA was used for whole genome sequencing.

### Method details

#### DNA isolation and genomic sequencing

For whole genome sequencing, blood samples were collected from one female Ossabaw pig (no. 2702, ‘Donna’, 2.5 years of age) housed at the DTU Ossabaw facility, Risø, Denmark. High quality DNA were isolated from 2x10^7^ peripheral blood mononuclear cells using Qiagen Genomic Tip 100/G long fragment DNA isolation kit. StLFR library and ONT libraries were constructed following the manufacturer’s protocols. Next, stLFR library was sequenced using the BGISEQ-500 platform, yielding ∼306 Gb paired-end short reads. The ONT library was sequenced using the GridION X5 platform, yielding ∼24 Gb long reads.

#### Genome assembly and annotation

Raw sequencing paired-end short reads were filtered using SOAPfilter (v2.2) (Luo et al., 2012) with parameters “-y -p -M 2 -f -1 -Q 10”. The software Supernova (v2.11) (Weisenfeld et al., 2017) was firstly used to assemble the Ossabaw pig genome with default parameters. Next, Redundans (Pryszcz and Gabaldón, 2016) was used to remove possible redundant heterozygous assembled sequences, after which stLFR_GapCloser was used for the first round of filling gaps based on short paired-end reads. Raw ONT long reads were firstly cut into ten portions (about 1Gb data per portion) then error-base correction was performed for each portion using 130 Gb (∼130X) short reads with running two rounds of Pilon (Walker et al., 2014) and next was used for the second round of filling gaps by using TGS-GapCloser (Xu et al., 2019). Finally, RaGOO (Alonge et al., 2019) was used to anchor assembled scaffolds to chromosomes using Duroc genome (NCBI accession: GCA_000003025.6) as a reference. Genomic repetitive sequences were annotated by combing homolog-based and *de novo* prediction methods. Firstly, transposable elements were identified using RepeatMasker (v4.0.5) (Tarailo-Graovac and Chen, 2009) and RepeatProteinMask (v4.0.5) against the Repbase database (Jurka et al., 2005) at the nuclear and protein levels, respectively. Secondly, RepeatModeler (v1.0.8) and LTR-FINDER (v1.0.6) (Xu and Wang, 2007) were used to carry out de novo prediction and construct a transposable element database, which was used to predict transposable element using RepeatMasker again. Thirdly, tandem repeats were predicted by using Tandem Repeat Finder (v4.0.7) (Benson, 1999). Prediction of protein-coding genes was also based on homolog and de novo evidences. For homolog prediction, protein sequences of six species including human (GCF_000001405.39), mouse (GCF_000001635.26), cattle (GCF_002263795.1), goat (GCF_001704415.1), horse (GCF_002863925.1) and pig (Duroc, GCF_000003025.6) were downloaded from NCBI database (Pruitt et al., 2011). Then these protein sequences were mapped to the assembled genome to identify candidate gene loci using BLAT (Kent, 2002) and Gene models were then predicted using GeneWise (v2.4.1) (Birney et al., 2004) based on the BLAT results with default parameters. For de novo prediction, we used Augustus (v3.1) (Keller et al., 2011) to predict the gene models based on the repeat-masked genome. Finally, EVidenceModeler (Haas et al., 2008) was used to integrate homolog and de novo prediction results to generate the final protein-coding gene set. BUSCO (v2.0) (Simao et al., 2015) and mammalia_odb9 lineage datasets were used to assess the quality of final gene set. To assign function each predicted gene, we mapped them to Kyoto Encyclopedia of Genes and Genomes (KEGG v84.0) (Kanehisa and Goto, 2000) , Swiss-Prot (v2017_09) (Boeckmann et al., 2003), TrEMBL (v2017_09) (Boeckmann et al., 2003) databases using BLASTp (v 2.2.26) (Altschul et al., 1990) with an *E*-value cutoff of 1×10^-5^. Gene domains and motifs were predicted using InterProScan (v5.16-55.0) (Apweiler et al., 2001) against ProDom (Bru et al., 2005), Pfam (Punta et al., 2012), SMART (Ponting et al., 1999), PANTHER (Mi et al., 2005), and PROSITE (Hulo et al., 2006). Gene ontology (GO) (Ashburner et al., 2000) terms were extracted from the InterProScan results. Short non-coding RNA genes were predicted using annotation from RFAM (Burge et al., 2013) and miRbase (Kozomara and Griffiths-Jones, 2010).

#### SNP calling and PSMC analysis

About 130 Gb clean short reads were mapped to the assembled Ossabaw genome using BWA-MEM (v0.7.12-r1039) (Li and Durbin, 2010) with default parameters, following which SAMtools (v0.1.19-44428cd) (Guindon et al., 2009) was used to convert SAM files to BAM format. “SortSam.jar” included in the Picard package (v1.54) was used to sort BAM files. Next, SNP calling were carried out by using two methods: 1)Local realignment was again carried out using RealignerTargetCreator and IndelRealigner in GATK v4.1.2 (McKenna et al., 2010) with default parameters. SNPs were identified using HaplotypeCaller and filtered using VariantFiltration with parameter “-filter-expression "QD < 2.0 || MQ < 40.0 || ReadPosRankSum < -8.0 || FS > 60.0" --filter-name LowQualFilter --genotype-filter-expression "DP < 5.0" --genotype-filter-name lt_5”. 2) The mpileup module in SAMtools and BCFtools (http://samtools.github.io/bcftools/bcftools.html) were also used to call SNPs with filtering parameters “-Q 20 -d 5”. Finally, we retained SNPs that were identified by both methods as the final SNPs set for further analysis. The estimation of historical effective population size were carried out using PSMC (v0.6.5-r67) (Li and Durbin, 2011). In details, firstly, diploid genome reference was constructed using “samtools mpileup -C50” and “vcfutils.pl vcf2fq -d 5 -D 1000”. Secondly, the demographic history was inferred using PSMC with parameters ‘-N25 -t15 -r5 -p 4+25∗2+4+6’ (Warren et al., 2017). SNPs calling and estimation of historical effective population size of the Duroc genome were estimated using the same method.

#### Gene family analysis

Coding and protein sequences of 8 non-pig species and 12 pig breeds (Table S16) used for gene family analysis were downloaded from NCBI database. Firstly, we filtered sequences according to the protein lengths with setting cutoff of 30 amino acid (aa). Secondly, we kept the longest transcript of every gene. Thirdly, we defined gene families with the TreeFam tool (Li et al., 2006). Gene family analyses were carried out among representative terrestrial mammals and different pig breeds, respectively. CAFE (Computational Analysis of gene Family Evolution, v2.1) (De Bie et al., 2006) was used to perform gene family expansion and contraction analysis with results from the TreeFam pipeline and the estimated divergence time (described below) between species as inputs and parameters “-p 0.01, -r 10000, -s” to search for the birth and death parameter (λ). Finally, *p*-values were calculated and gene families with *p*-values <0.05 were defined as expanded/contracted significantly.

#### Construction of phylogenetic tree and estimation of divergence time

Single copy orthologous genes (SCOGs) obtained from TreeFam pipeline were used for construct phylogenetic trees. These SCOGs were aligned using software MUSCLE (Edgar, 2004) with default parameters. All of these gene alignment results were linked to a super-gene for each species. Then phylogenetic trees were constructed using both maximum likelihood and Bayesian methods with a GTR+gamma model. Finally, the PAML mcmctree program (Yang, 2007; Yang and Rannala, 2006) was used to estimate divergence times among species. When estimating divergence times, we used seven time points obtained from TimeTree database (http://www.timetree.org) for calibration. These times points include *Monodelphis domestica*- *Homo sapiens* (∼151-166 MYA), *Homo sapiens*- *Mus musculus* (∼85-97 MYA), *Homo sapiens*- *Canis lupus familiaris* (∼91-101 MYA), *Canis lupus familiaris*- *Equus caballus* (∼70.2-79.0 MYA), *Camelus dromedarius*- *Equus caballus* (∼76-82 MYA), *Camelus dromedarius*- *Bos taurus* (∼62-68 MYA) and *Bos taurus*- *Capra hircus* (∼22.17-29.00 MYA)

#### Identification of positively selected genes.

PRANK (Löytynoja, 2014) was used for carrying out alignments of coding sequences of all SCOGs and gBlocks (Talavera and Castresana, 2007) was used for removing poorly aligned sites. High-quality alignments were then filtered to estimate the ratios (ω=d_N_/d_S_) of nonsynonymous nucleotide substitutions (d_N_) to synonymous nucleotide substitutions (d_S_) for these genes in the target species (ω_0_), background branches (ω_1_) or all branches (ω_2_) using the codeml program with an improved branch-site model (Zhao et al., 2010) (model = 2, NSsites = 2) and the maximum likelihood method in the PAML package (Yang, 2007) . LRTs (likelihood ratio tests) were performed and *P*-values were computed based on the Bayes Empirical Bayes (BEB) method (Zhang et al., 2005) which can avoid an excessive false positive rate (Suzuki and Nei, 2004), and genes with *P*-values less than 0.05 were treated as candidates of positive selection.

#### Identification SVs compared to Duroc

Structure variations (SV) were identified with two pathways. 1)For inversions: LastZ (v1.1) (Harris, 2007) was used for whole genome alignment with parameters “T=2 C=2 H=2000 Y=3400 L=6000 K=2200 --format=axt”. Then “axtChain”, “chainMergeSort”, “chainPreNet”, “chainNet”, “netSyntenic”, “netToAxt”, “axtSort” and “axtToMaf” from UCSC (http://hgdownload.cse.ucsc.edu/admin/exe/) were used ordinally to deal with alignment results. 2)For insertions and deletions (InDels): Smartie-sv (Kronenberg et al., 2018) was used to map contigs of Ossabaw genome to Duroc genome with default parameters.

#### Comparative transcriptome analysis

Raw RNA sequencing reads of 13 Ossabaw pig samples with accession numbers (SRR8844240, SRR8844241, SRR8844242, SRR8844243, SRR8844244, SRR8844245, SRR8844246, SRR8844255, SRR8844256, SRR8844257, SRR8844258, SRR8844259 and SRR8844260) were download from NCBI (SRA) database. These samples include two groups: Heart Healthy diet (high in unsaturated fat, unrefined grain, fruits/vegetables [HHD]) and Western diet (high in saturated fat, cholesterol, refined grain [WD]) (Walker et al., 2019). Raw sequencing data was filtered using SOAPnuke (v1.6.5) (Chen et al., 2017) with parameters “-l 10 -q 0.2 -n 0.05 -5 1 -i -Q 2 -5 1 -c 0 -1”. Clean sequencing data was mapped to the assembled Ossabaw genome using bowtie2 (Langmead and Salzberg, 2012) with parameters “--sensitive --dpad 0 --gbar 99999999 --mp 1,1 --np 1 --score-min L,0,-0.1 -p 16 -k 200”. Then RSEM (v1.2.31) (Li and Dewey, 2011) was used to calculate each gene expression level (FPKM).

#### Gene enrichment

Enrichment analysis of genes was conducted using GOstat and enrichKEGG (Beissbarth and Speed, 2004; Huang et al., 2009).

### Quantification and statistical analysis

Quantification and statistical analyses used in the genome sequencing and assembly, genome quality assessment, evolutionary analysis and comparative transcriptome analysis can be found in the relevant sections of the method details.

## Data Availability

Assembled genomes and raw sequencing data that support the findings of this study are publicly accessible and have been deposited into CNGB Sequence Archive (CNSA) (Guo et al., 2020) of China National GeneBank DataBase (CNGBdb) (Chen et al., 2020), https://db.cngb.org/cnsa/ with accession number CNP0001681. This paper does not report original code. Any additional information will be shared by the lead contact upon reasonable request.

## Supplemental information

Document S1. Figures S1–S14 and Tables S1–S12, S14, S16, S18, S20, and S22–S24

Table S13. KEGG enrichment of genes with heterozygous loci, related to Figure 1

Table S15. List of NAFLD pathway genes, related to Figure 2

Table S17. List of 196 positively selected genes, related to Figure 2

Table S19. List of 536 bins with high density genes and SNPs, related to Figure 3

Table S21. GO enrichment analysis of candidate fast evolved genes, related to Figure 3
