# Supplementary material for: The genome of the naturally evolved obesity-prone Ossabaw miniature pig
Source: iScience. 2021 Sep 3;24(9):103081. doi: 10.1016/j.isci.2021.103081 (PMC8455653; doi:10.1016/j.isci.2021.103081)
Supplement: Document S1. Figures S1–S14 and Tables S1–S12, S14, S16, S18, S20, and S22–S24 [file mmc1.pdf]

iScience, Volume *24*

Supplemental information

The genome of the naturally evolved obesity-prone Ossabaw miniature pig

Yaolei Zhang, Guangyi Fan, Xin Liu, Kerstin Skovgaard, Michael Sturek, and Peter M.H. Heegaard

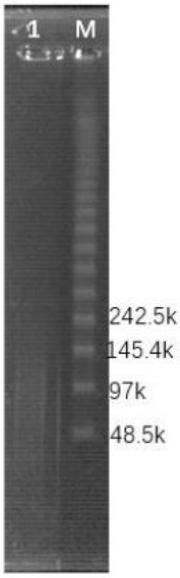

Figure S1. DNA quality assessment by using pulsed field gel electrophoresis, Related to Figure 1.

Parameter: "Run time: 16h; Cooling temperature: 14 centigrade; Volt/s: 6V/cm; Initial switch time: 0.22s; Final switch time: 44.69s".

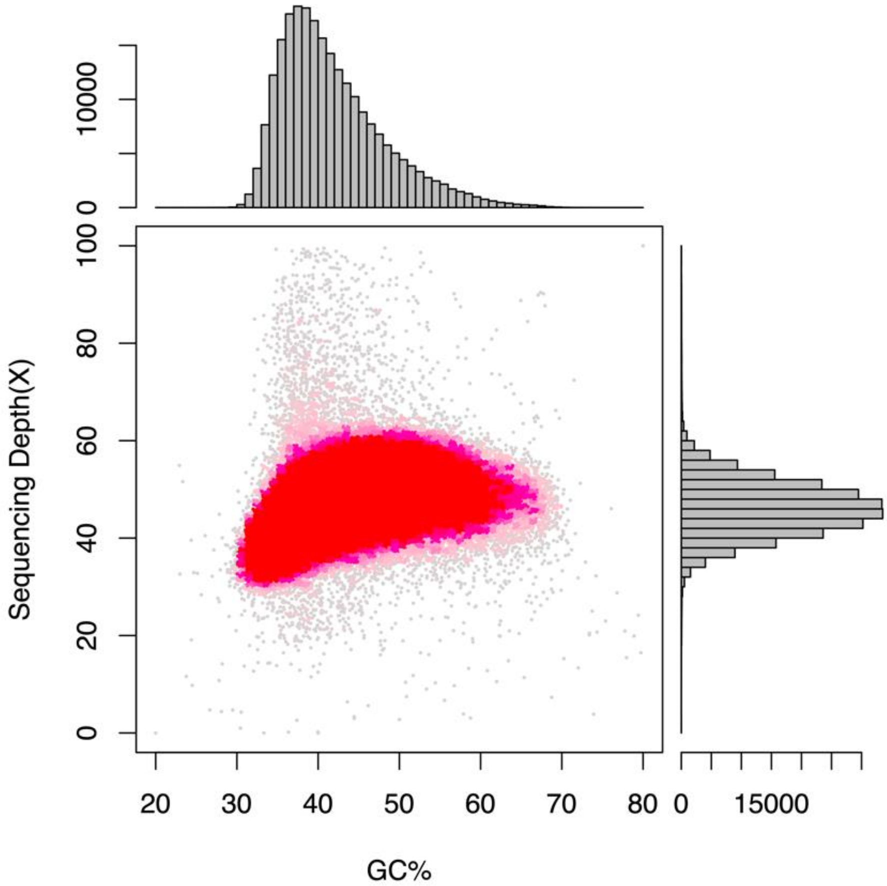

Figure S2. GC content and sequencing depth of Ossabaw genome with 10 Kb bins using ~130 Gb short reads, Related to Figure 1.

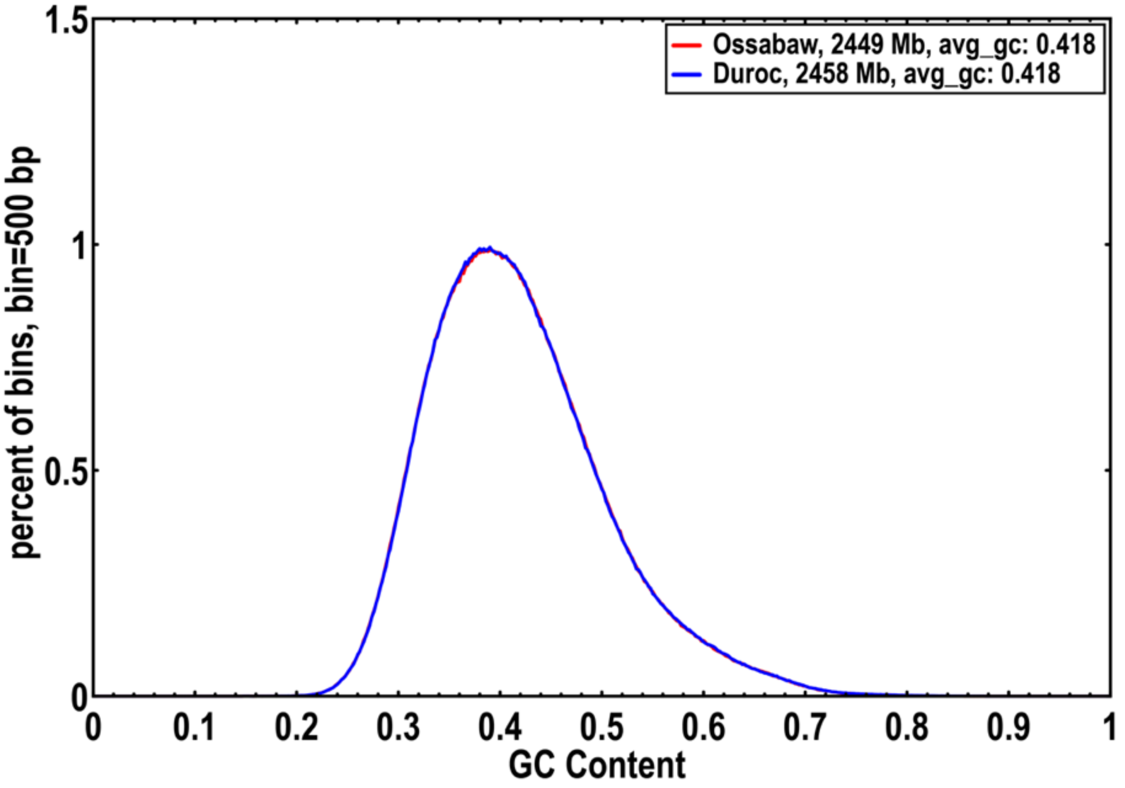

Figure S3. GC content distribution of Ossabaw and Duroc genomes with 500 bp bins, Related to Figure 1.

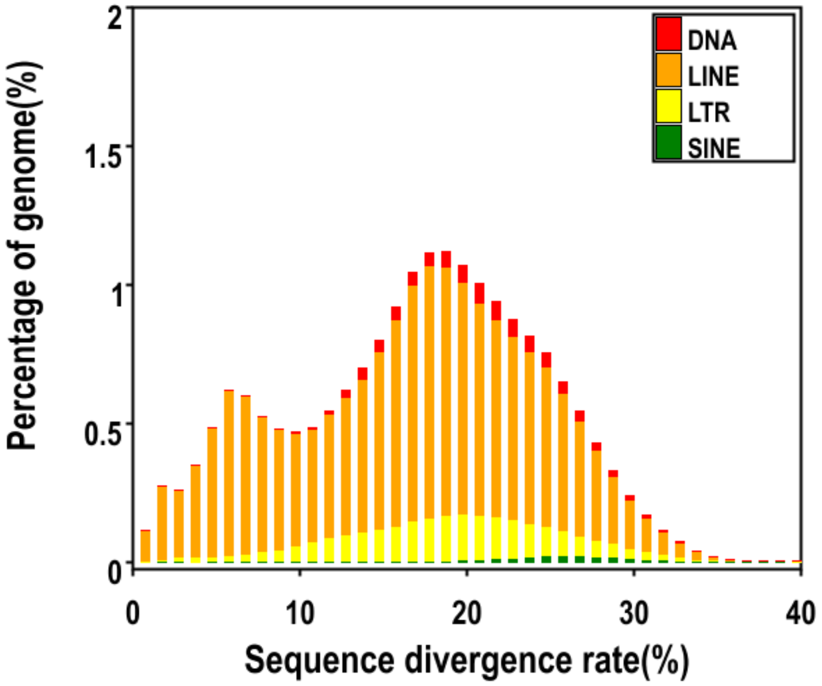

Figure S4. Sequence divergence distribution of predicted TEs in ossabaw pig genome compared to Repbase database, Related to Figure 1.

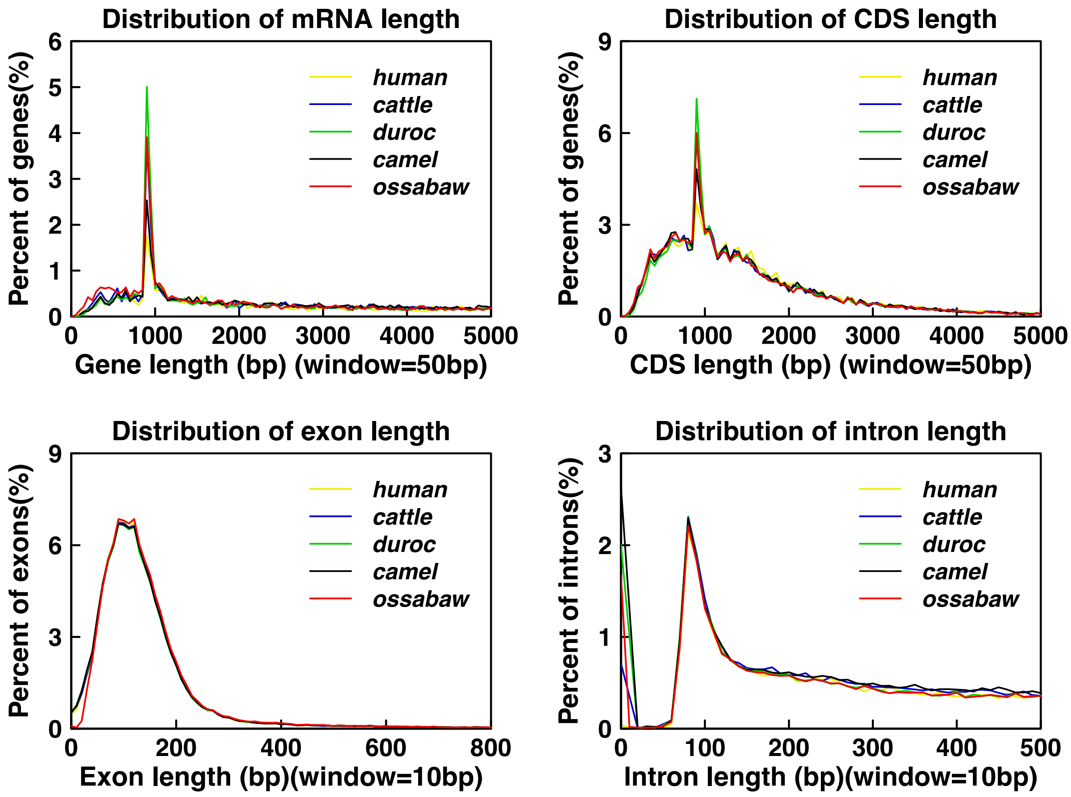

Figure S5. Gene, CDS (coding sequences), exon and intron length distribution of Ossabaw pig predicted genes compared to Duroc, Human, Cattle and Camel, Related to Figure 1.

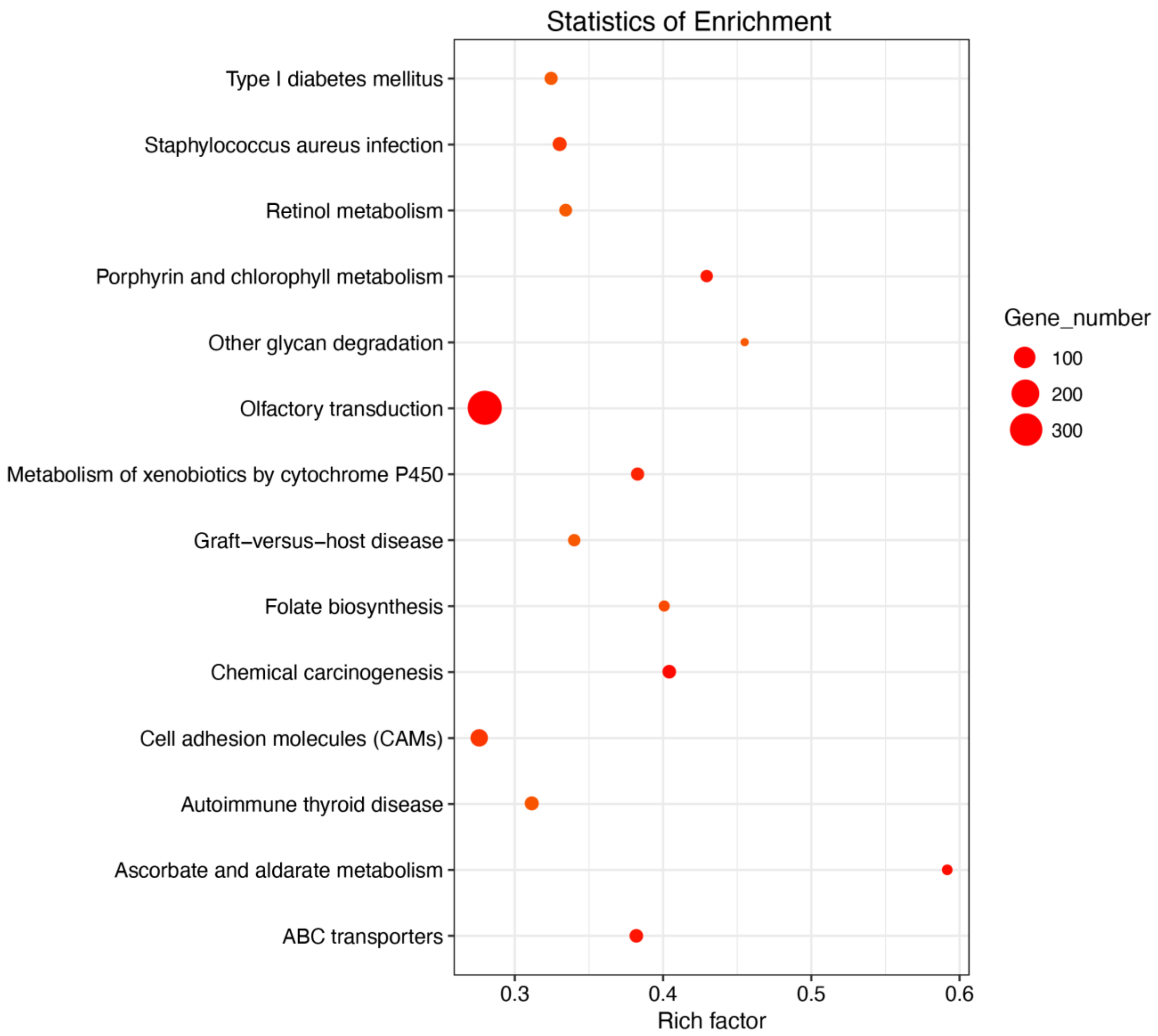

Figure S6. Top 20 pathways (based on q-value increase) of 1,811 genes with heterozygous SNPs, Related to Figure 1. All *q*-values are below 0.05

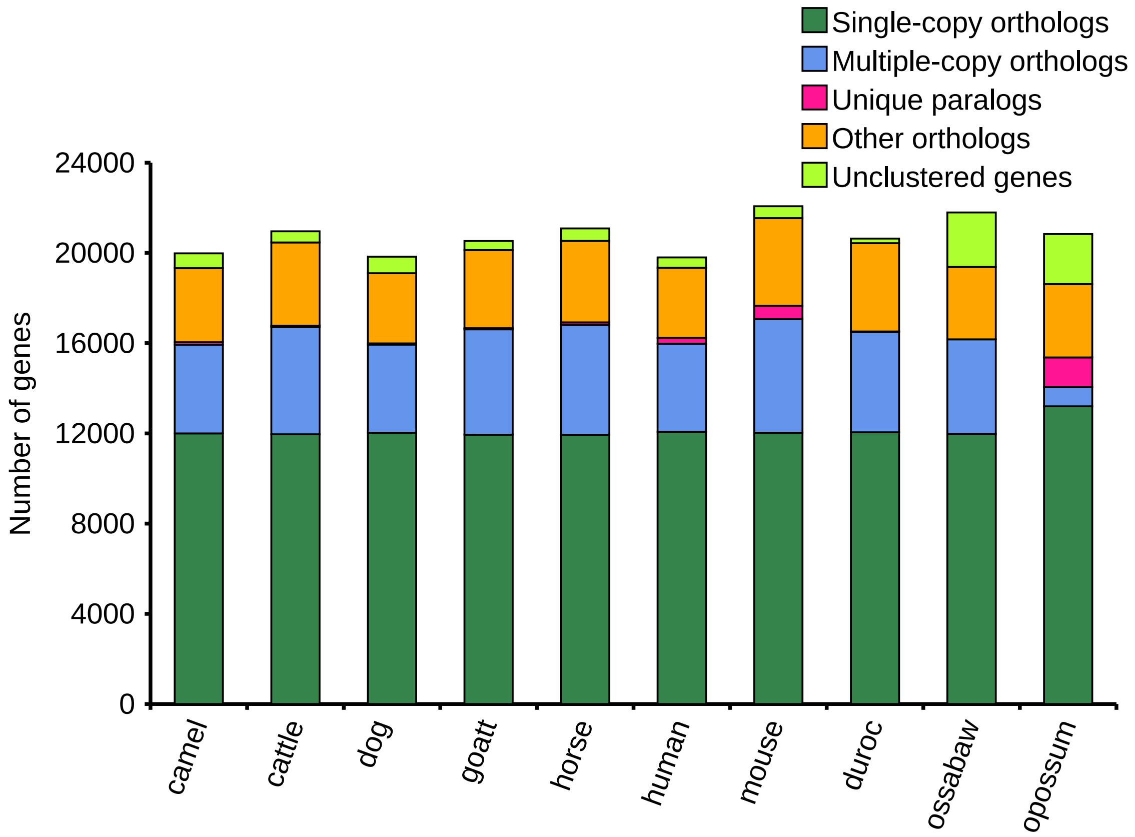

Figure S7. Gene family clusters in ten representative mammalian species, Related to Figure 2.

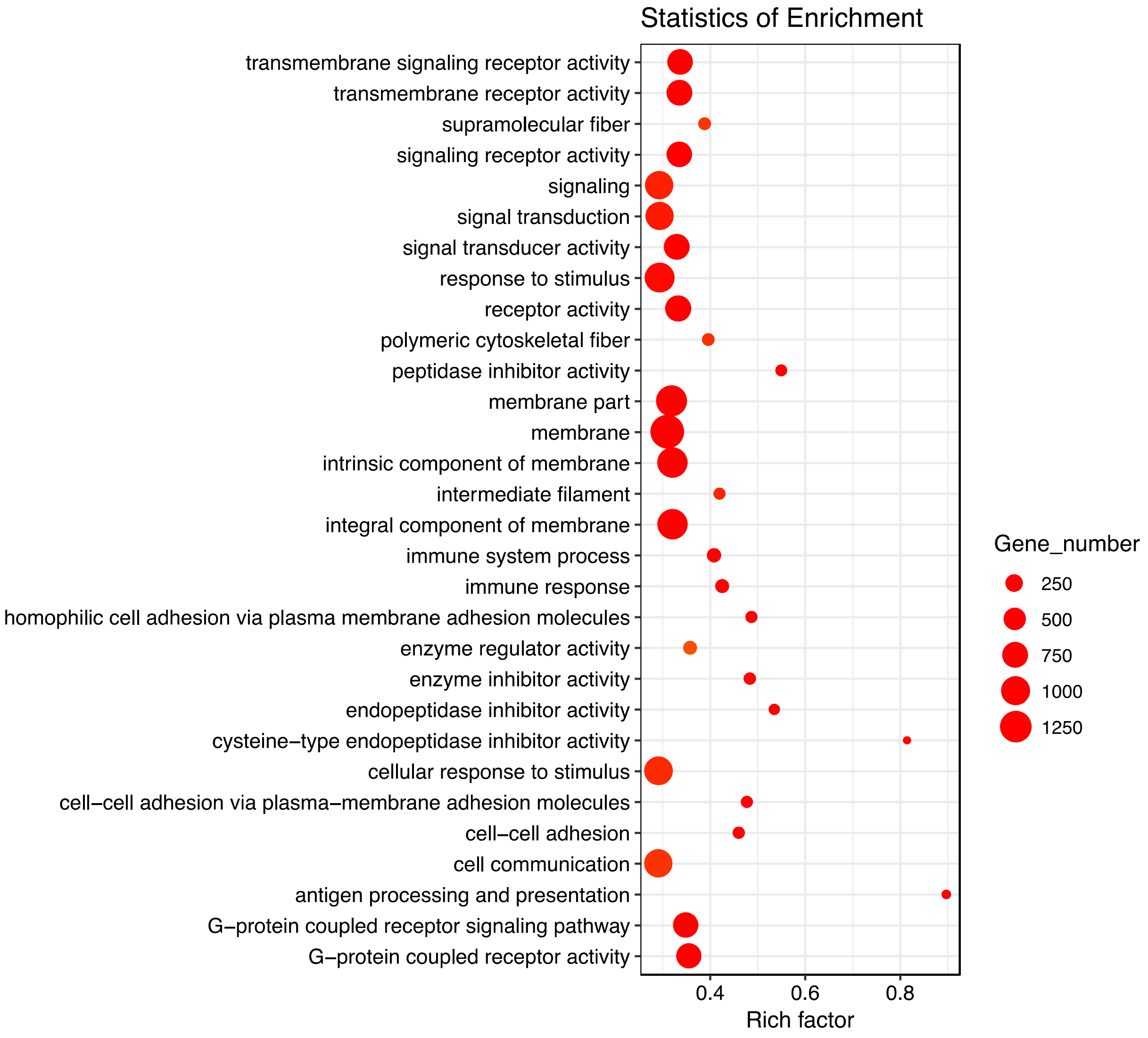

Figure S8. Top 30 enriched GO terms of fast evolved candidate genes, Related to Figure 2. All enrichment *q*-values are below 0.05

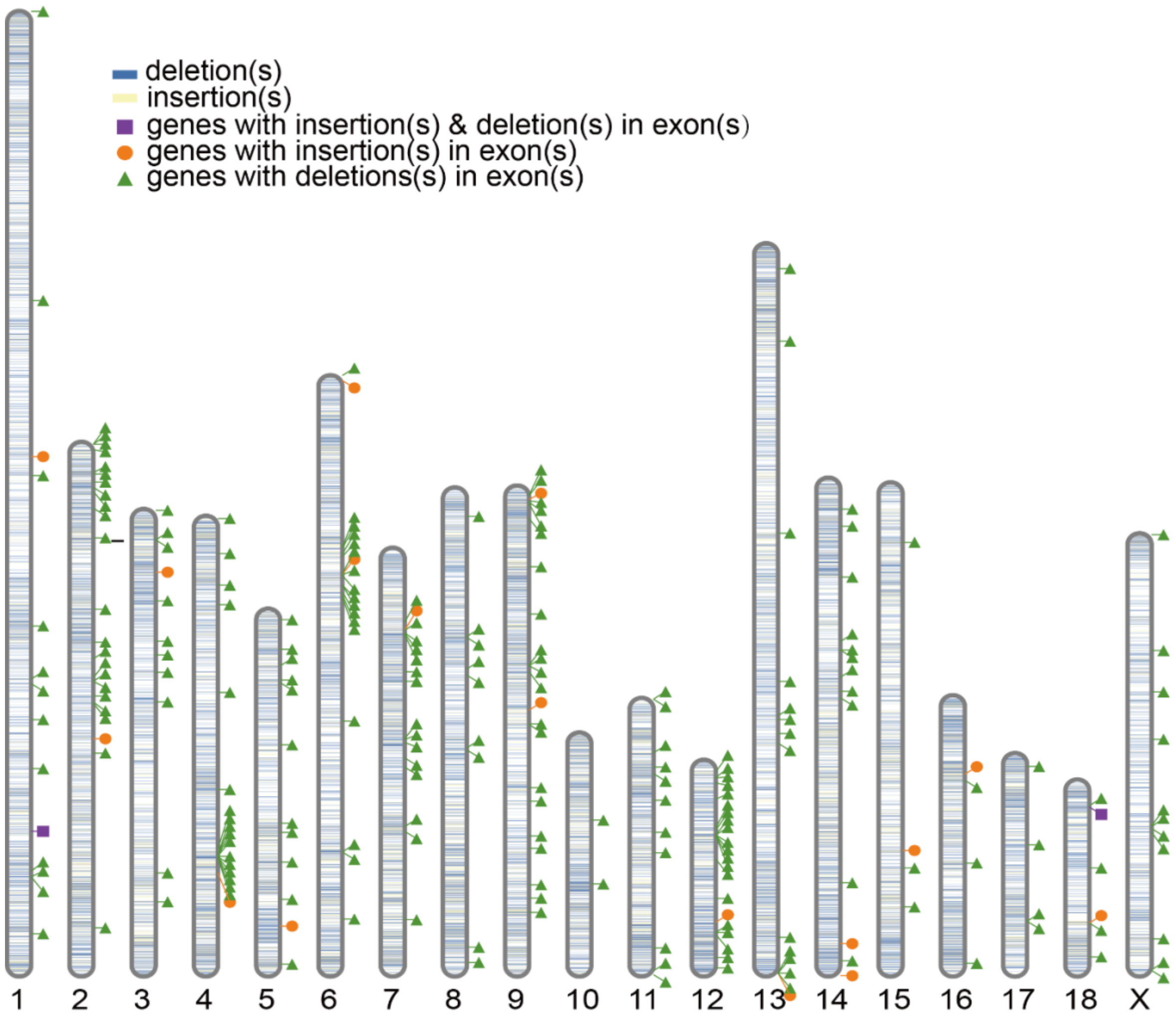

Figure S9. Indels distributions and genes with indels located in exon regions, Related to Figure 3.

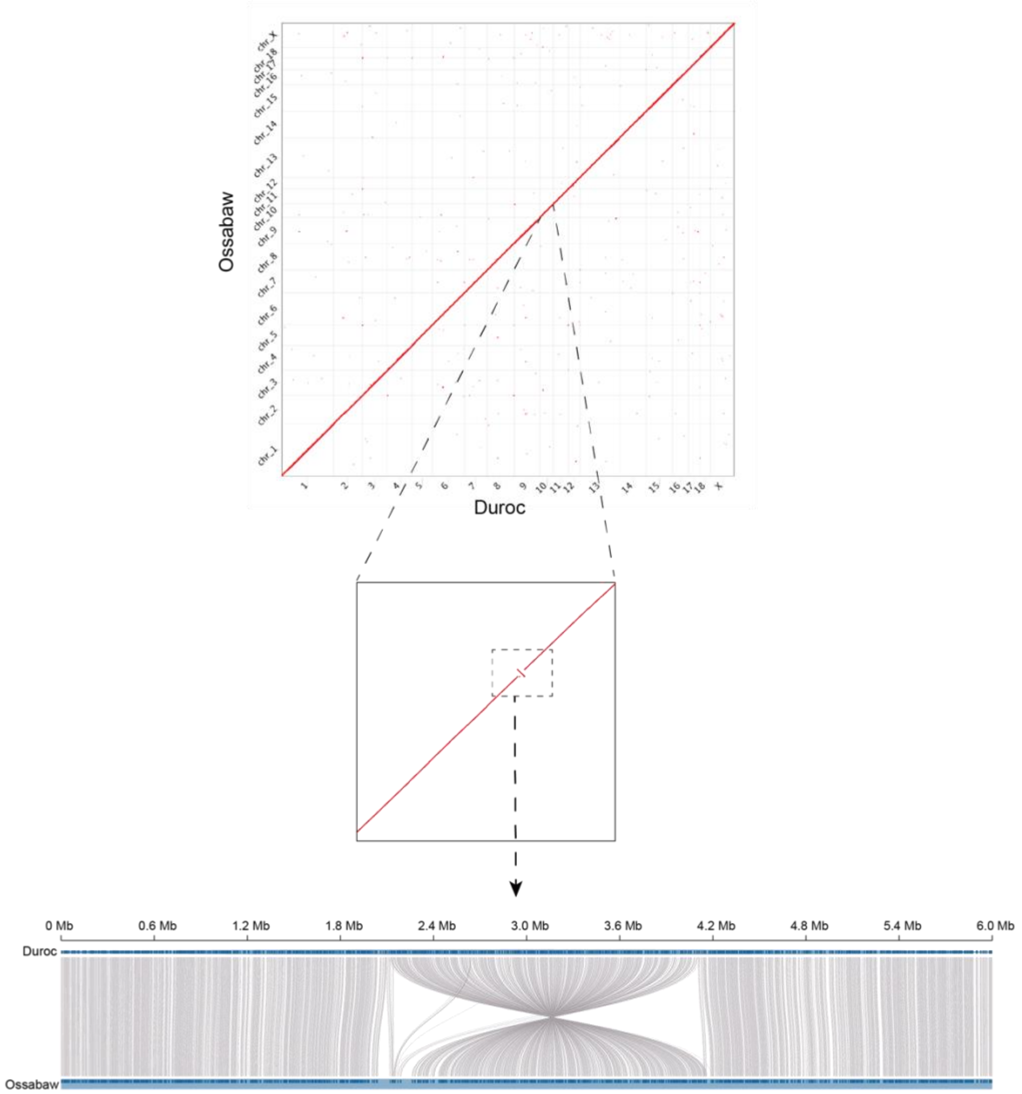

Figure S10. Inversion between Ossabaw and Duroc genome on chromosome 10, Related to Figure 3.

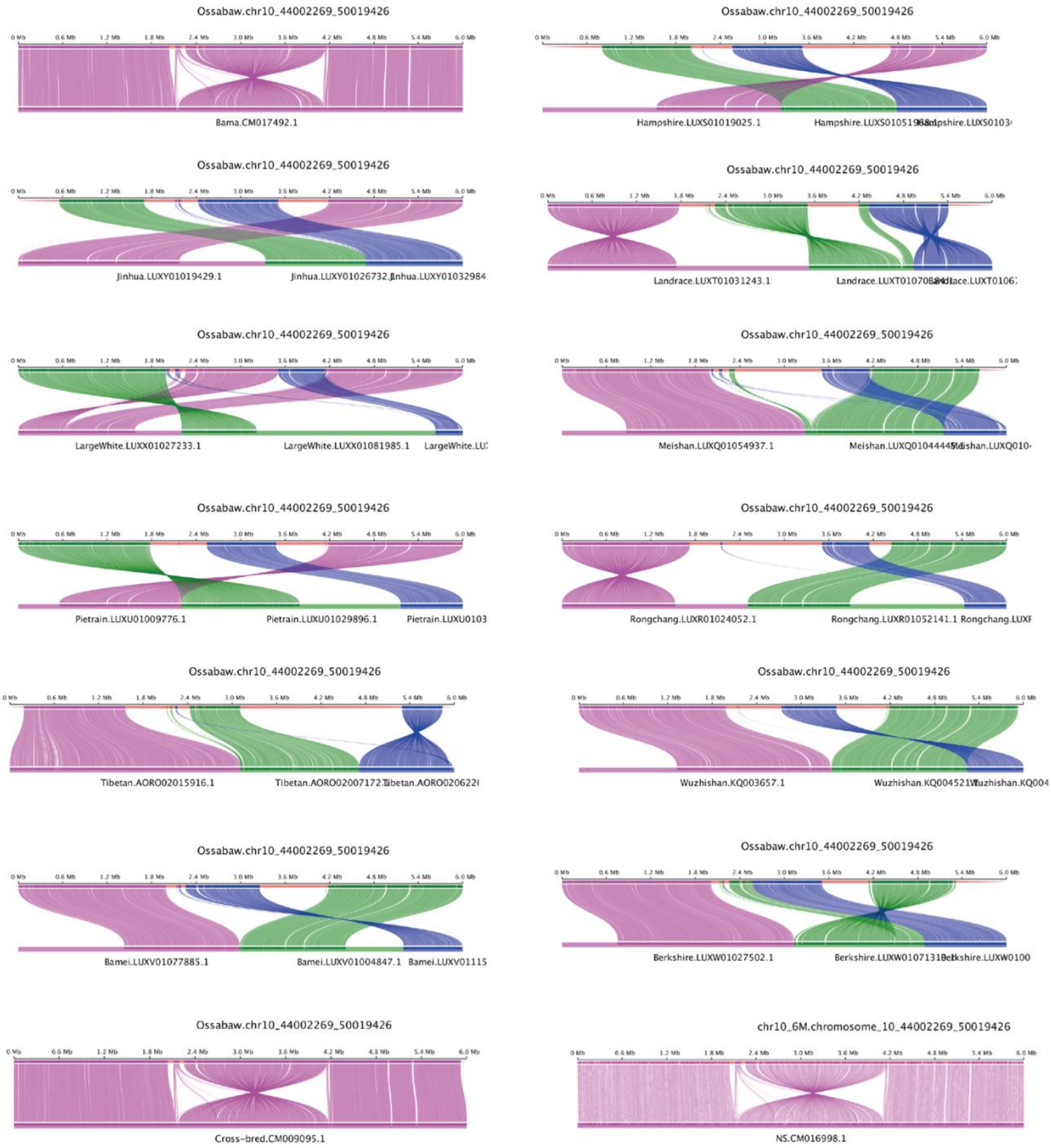

Figure S11. Comparison of the ~2Mb inversion with 14 other pig breeds. Related to Figure 3.

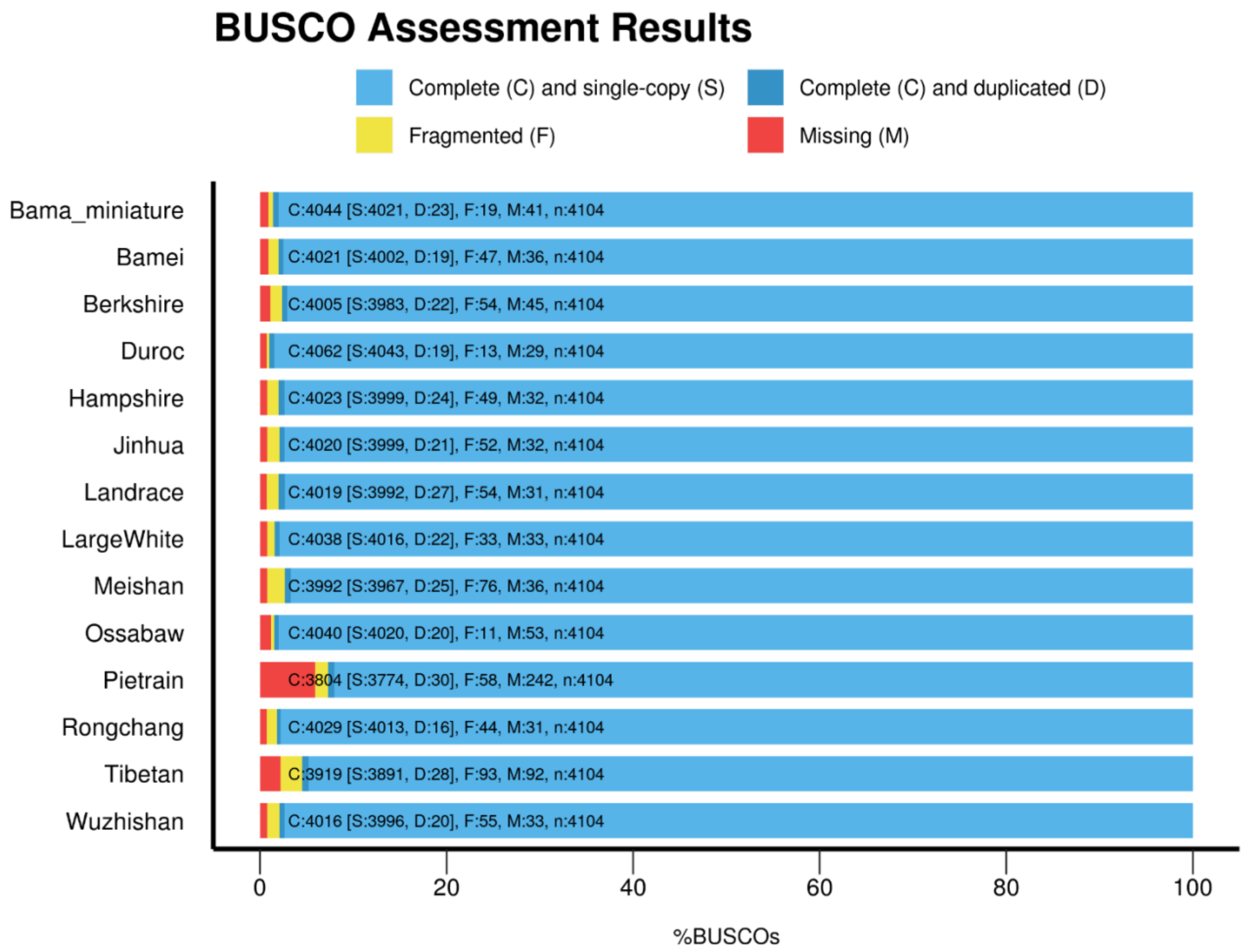

Figure S12. BUSCO assessment of genes of 14 pig breeds, Related to Figure 4.

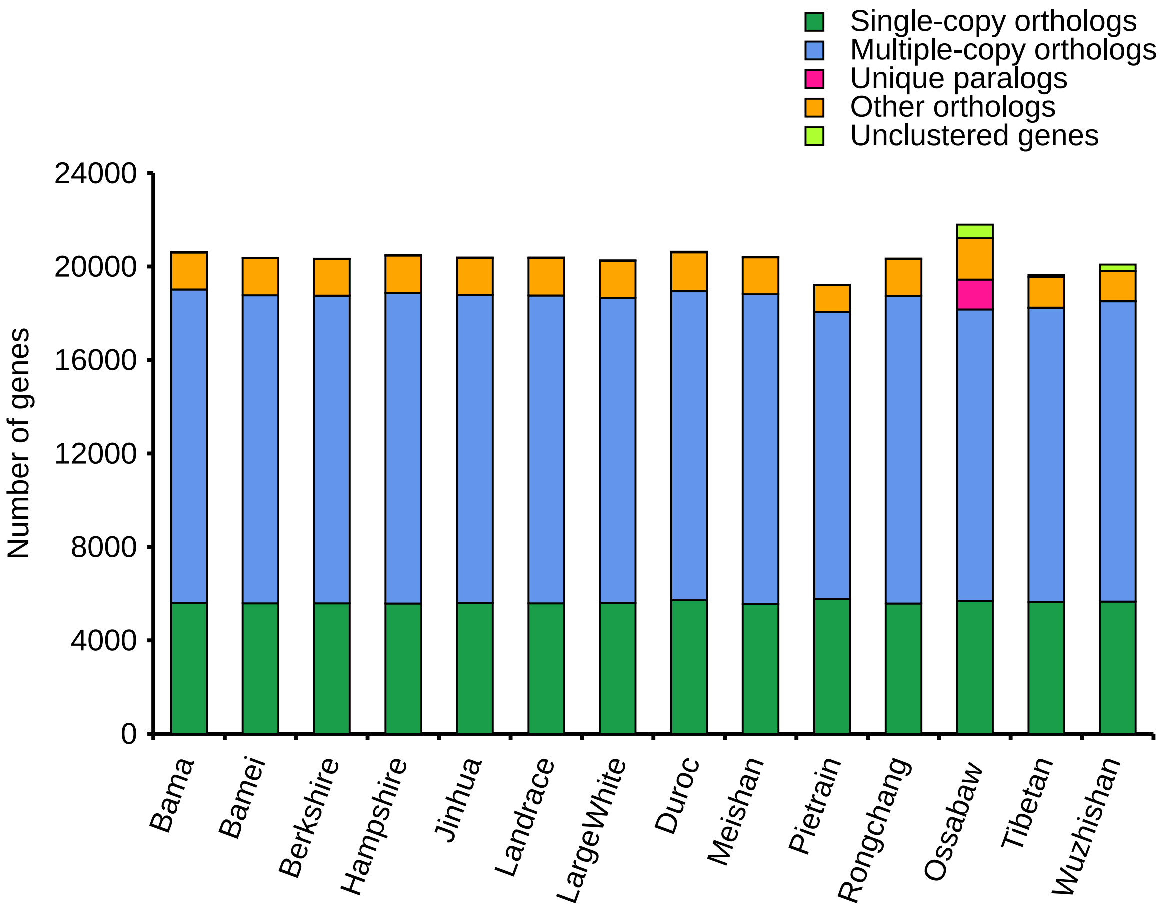

Figure S13. Gene family clusters among 14 pig breeds, Related to Figure 4.

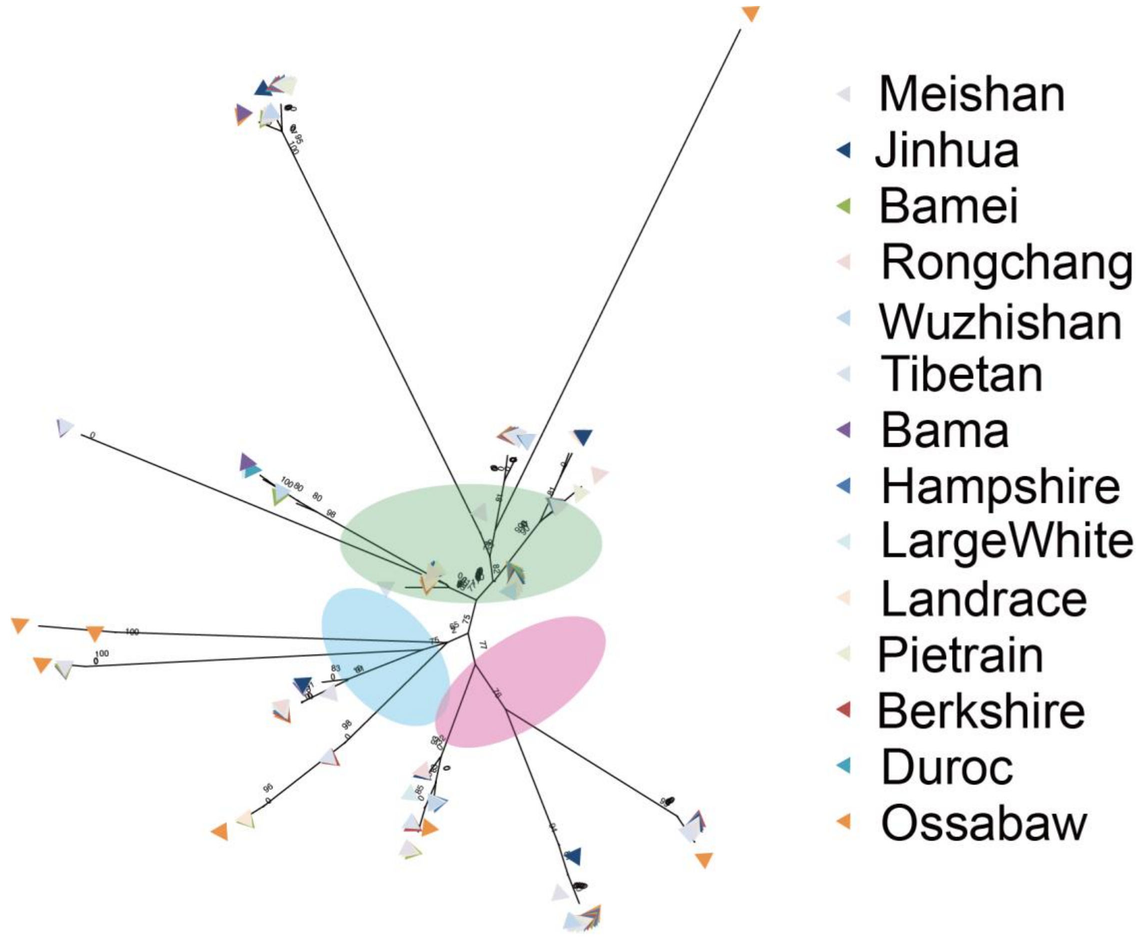

Figure S14. Expansion of Cytochrome c gene, Related to Figure 4.

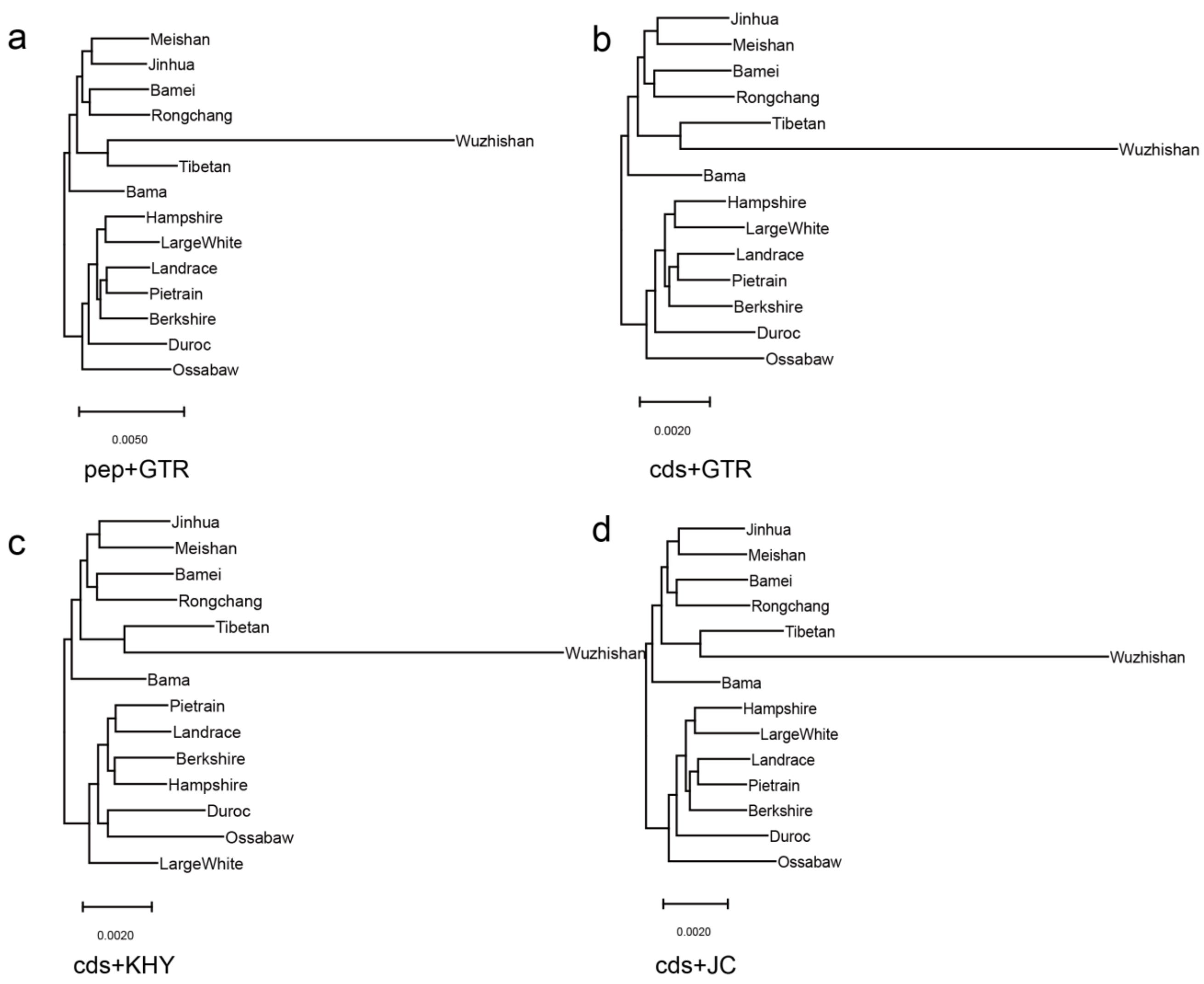

Figure S15. Phylogenetic tree of 14 pig breeds by using different models, Related to Figure 4. pep+GTR: Tree constructed using protein sequences with GTR model. cds+GTR, cds+HKY and cds+JC: Trees constructed using coding sequences (nucleotide sequences) with GTR, HKY and JC models respectively.

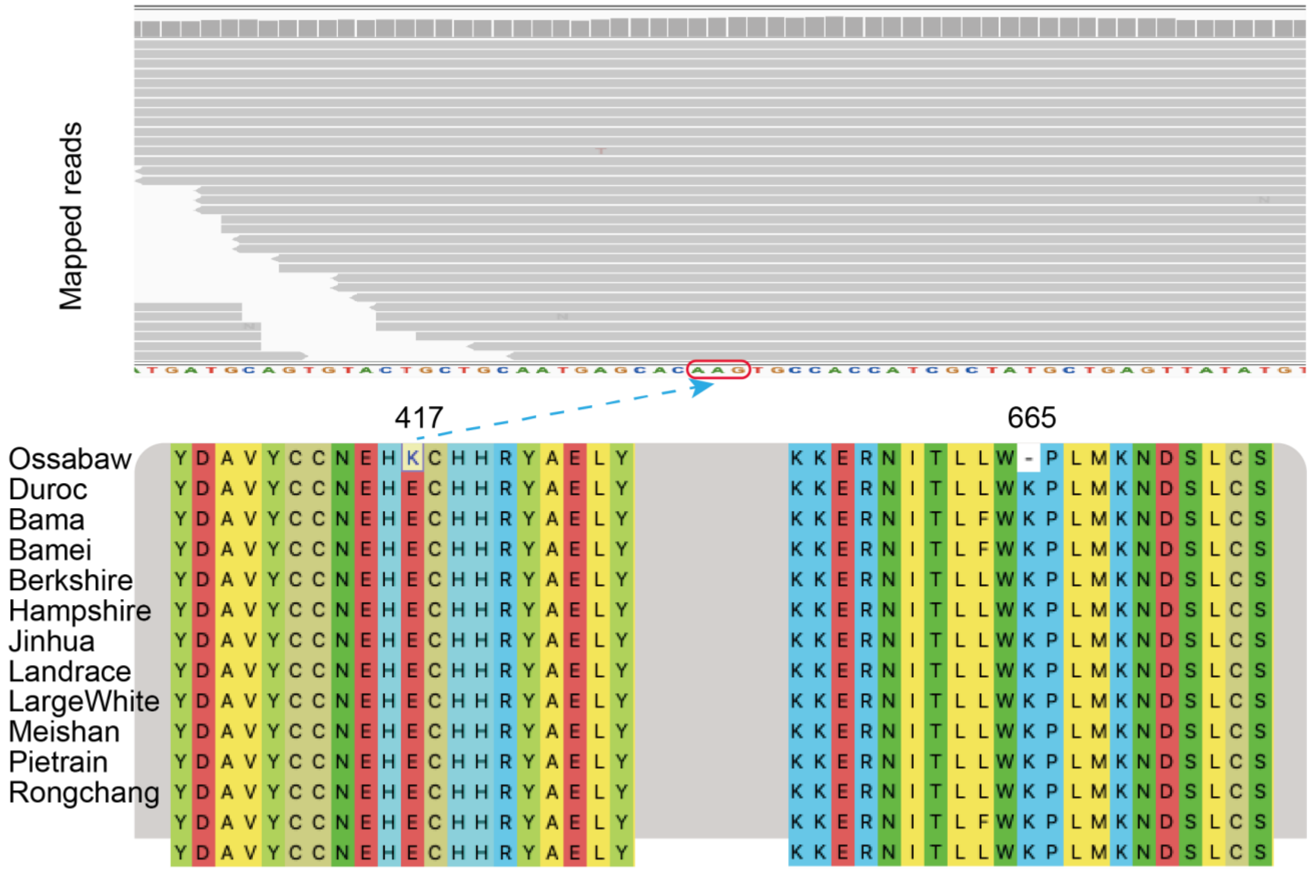

Figure S16. Reads mapping of first positively selected site of *LEPR* gene, Related to Figure 4.

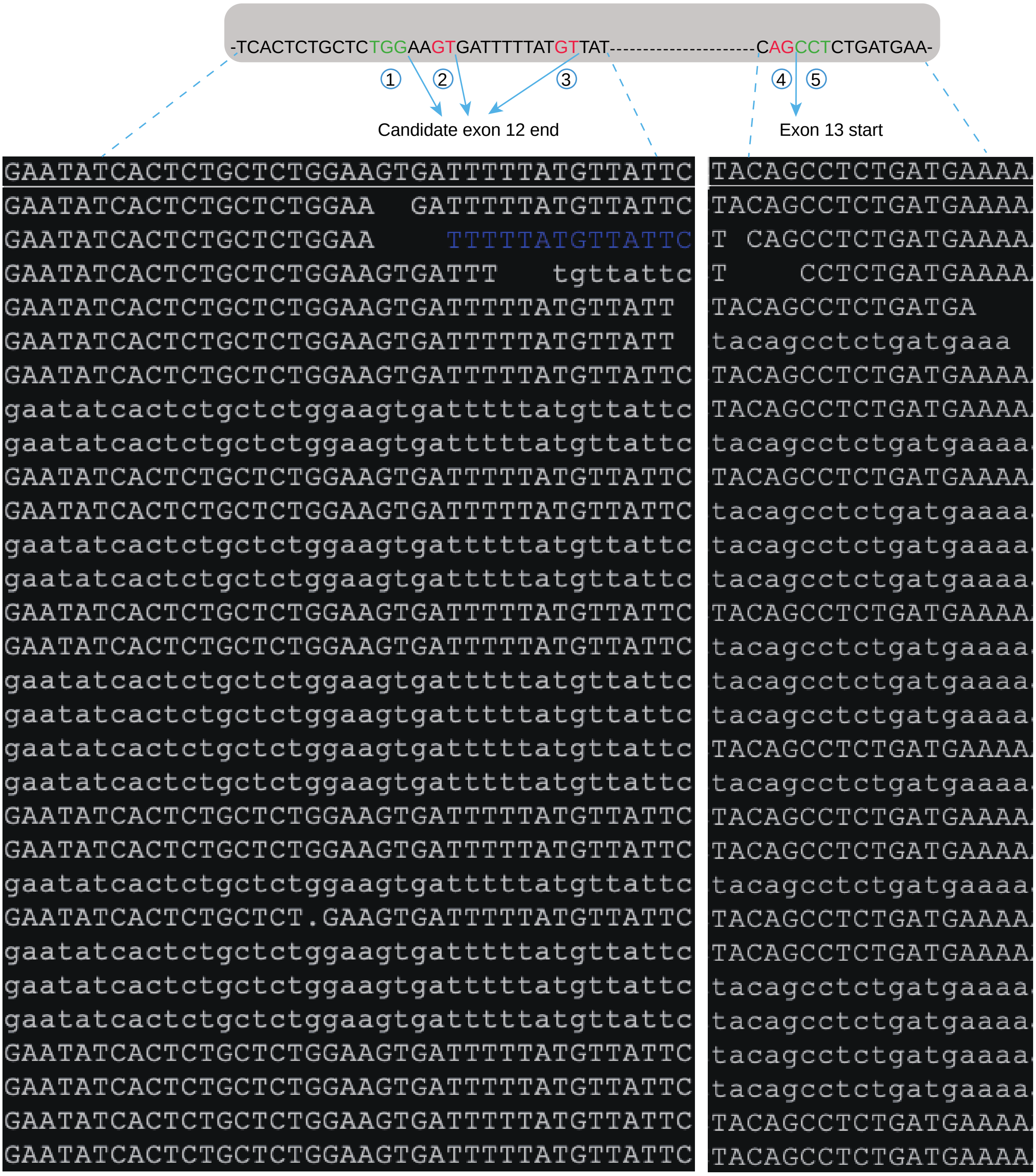

Figure S17. Reads mapping of second positively selected site of *LEPR* gene, Related to Figure 4.

Supplementary Tables:

Table S1. Statistics of sequencing raw data for Ossabaw pig, Related to Figure 1

Sequencing platform	Libraries	Total Data (Gb)	Coverage (X)
BGISEQ-500	stLFR	305.83	122.33
GridION X5	ONT	24.18	9.67
Total	\	330.01	132.00

Table S2. Genome assembly summary of Ossabaw pig (V4), Related to Figure 1.

V4	Scaffold	Contig
Total length of (bp):	2,454,571,501	2,452,057,832
Gap number (bp):	2,513,669	0
Average length (bp):	214,017.92	198,274.27
N50 Length (bp):	22,308,774	6,028,600
N90 Length (bp):	5,113,492	1,229,594
Maximum length (bp):	79,055,197	36,004,294
GC content is (%):	41.91	41.91

Table S3. Comparison of Ossabaw pig genome with published other breed pig genomes, Related to Figure 1.

Breed	NCBI accession	Total length(bp)	Total ungapped length(bp)	Scaffold N50(bp)	Contig N50(bp)
Meishan	GCA_017957985.1	2,510,037,796	2,493,974,684	138,968,154	51,011,532
Duroc	GCA_000003025.6	2,501,912,388	2,472,047,747	88,231,837	48,231,277
Duroc (excluding Y)	GCA_000003025.6	2,458,364,560	2,456,479,391	138,966,237	41,891,233
Cross bred	GCA_006511355.2	2,755,438,182	2,623,130,238	131,458,098	6,372,407
**Ossabaw(V4)**	/	**2,454,571,501**	**2,452,057,832**	**22,308,774**	**6,028,600**
**Ossabaw(V5)**	/	**2,455,685,401**	**2,452,057,832**	**140,684,285**	**6,028,600**
NS	GCA_006511355.2	2,479,330,878	2,449,733,784	142,372,574	594,997
Bama miniature	GCA_007644095.1	2,491,053,062	2,491,053,062	140,438,739	1,009,524
Hampshire	GCA_001700165.1	2,437,109,612	2,418,011,428	1,550,023	102,417
Jinhua	GCA_001700295.1	2,453,702,738	2,433,032,022	1,478,908	95,227
Berkshire	GCA_001700575.1	2,434,706,773	2,414,739,650	1,655,397	94,651
LargeWhite	GCA_001700135.1	2,457,907,103	2,430,896,979	2,441,555	88,831
Landrace	GCA_001700215.1	2,440,981,886	2,420,570,845	1,407,841	88,142
Pietrain	GCA_001700255.1	2,438,316,299	2,415,062,022	1,663,542	80,611
Rongchang	GCA_001700155.1	2,459,027,728	2,429,730,895	2,325,000	79,093
Bamei	GCA_001700235.1	2,460,755,741	2,433,636,520	1,529,027	70,893
Meishan	GCA_001700195.1	2,467,495,771	2,467,495,771	1,248,180	63,263
Tibetan	GCA_000472085.2	2,437,742,524	2,379,878,366	861,885	57,199
Wuzhishan	GCA_000325925.2	2,508,912,457	2,453,484,489	5,853,977	31,939
Ellegaard Gottingen minipig	GCA_000331475.1	2,358,017,222	2,358,017,222	22,008	22,008
Goettingen	GCA_001292865.1	2,611,360,562	2,043,668,444	153,651,326	17,259

Note: The Duroc reference genome is based on a female Duroc sow with a Y chromosome added from "WTSI_X_Y_pig V2 (https://www.ncbi.nlm.nih.gov/assembly/GCA_900119615.2/#/st)".

Table S4. Evaluation of base accuracy by calling SNPs and INDELs in Ossabaw pig genome, Related to Figure 1.

	Heterozygous SNPs	Homozygous SNPs	Heterozygous INDELs	Homozygous INDELs	Base inaccuracy
Number	4,039,107	4,298	951,643	1,456	0.0002%

Table S5. Length of assembled 19 chromosomes of Ossabaw compared to Duroc, Related to Figure 1.

Chromosome ID Ossabaw/Duroc	Length (bp) Ossabaw/Duroc	Percentage (%) (Ossabaw/Duroc)
chromosome_1/ Chromosome_1	278,675,875/274,330,532	101.58
chromosome_2/ Chromosome_2	151,395,492/151,935,994	99.64
chromosome_3/ Chromosome_3	135,322,977/132,848,913	101.86
chromosome_4/ Chromosome_4	132,404,064/130,910,915	101.14
chromosome_5/ Chromosome_5	107,929,519/104,526,007	103.26
chromosome_6/ Chromosome_6	173,403,329/170,843,587	101.50
chromosome_7/ Chromosome_7	122,493,303/121,844,099	100.53
chromosome_8/ Chromosome_8	140,684,285/138,966,237	101.24
chromosome_9/ Chromosome_9	140,013,607/139,512,083	100.36
chromosome_10/ Chromosome_10	72,724,907/69,359,453	104.85
chromosome_11/ Chromosome_11	80,030,521/79,169,978	101.09
chromosome_12/ Chromosome_12	62,094,181/61,602,749	100.80
chromosome_13/ Chromosome_13	209,947,648/208,334,590	100.77
chromosome_14/ Chromosome_14	143,392,520/141,755,446	101.15
chromosome_15/ Chromosome_15	141,751,623/140,412,725	100.95
chromosome_16/ Chromosome_16	80,762,977/79,944,280	101.02
chromosome_17/ Chromosome_17	64,016,008/63,494,081	100.82
chromosome_18/ Chromosome_18	56,7383,42/55,982,971	101.35
chromosome_X/ Chromosome_X	128,445,696/125,939,595	101.99

Table S6. BUSCO assessment of assembled Ossabaw genome using mamalia_odb9 database, Related to Figure 1.

\	Gene number	Percentage (%)
Complete BUSCOs (C)	3,736	91.0
Complete and single-copy BUSCOs (S)	3,716	90.5
Complete and duplicated BUSCOs (D)	20	0.5
Fragmented BUSCOs (F)	206	5.0

Missing BUSCOs (M)	162	4.0
Total BUSCO groups searched	4,104	100.0

Table S7. Summary of transposable elements in Ossabaw genome, Related to Figure 1.

	Repbase TEs		TE proteins		De novo		Combined TEs		
Type	Length (bp)	% in genome	Length (bp)	% in genome	Length (bp)	% in genome	Length (bp)	% in genome	AL (bp)
DNA	20260987	0.826854	266596	0.01088	5057900	0.206414	24052258	0.981577	282.75
LINE	389704840	15.903918	188627423	7.697916	828809784	33.823861	883295085	36.047415	422.19
SINE	5198497	0.212152	0	0	36524	0.001491	5231832	0.213512	134.60
LTR	62177557	2.537476	980363	0.040009	8134913	0.331987	66140793	2.699216	366.20
Other	360	0.000015	0	0	0	0	360	0.000015	95.00
Unknown	0	0	0	0	111489	0.00455	111489	0.00455	828.00
Total	476857263	19.460622	189871626	7.748692	838716403	34.228152	936577450	38.221877	/

Note: DNA: DNA Transposons; LINE: Long Interspersed Nuclear Elements; SINE: Short Interspersed Nuclear Elements; LTR: Long Terminal Repeat.

Table S8. Classes of non-coding RNAs in the Ossabaw genome, Related to Figure 1.

Type	Number	Average length(bp)	Total length(bp)	% of genome
miRNA	825	78.5	64,759	0.0026
tRNA	4,320	75.68	326,923	0.0133
rRNA	462	100.01	46,206	0.0019
snRNA	1,608	113.35	182,274	0.0074

Table S9. BUSCO assessment of predicted genes using ammalia_odb9 database, Related to Figure 1.

\	Gene number	Percentage (%)
Complete BUSCOs (C)	4,040	98.5
Complete and single-copy BUSCOs (S)	4,020	98.0
Complete and duplicated BUSCOs (D)	20	0.5
Fragmented BUSCOs (F)	11	0.3
Missing BUSCOs (M)	53	1.2
Total BUSCO groups searched	4,104	100.0

Table S10. Summary of function annotation of predicted genes in Ossabaw genome, Related to Figure 1.

\	Total genes	Swissprot	KEGG	TrEMBL	Interpro	GO	All annotated
Gene Number	21,794	20,016	18,173	21,363	18,955	14,267	21,382
Percentage	100%	91.85%	83.39%	98.02%	86.97%	65.46%	98.11%

Table S11. Information of downloaded NCBI Duroc data, Related to Figure 1.

	NCBI accession	Data size (Gb)
Duroc 1	SRR5357801, SRR5357802	28.02
Duroc 2	SRR7142641, SRR7142642, SRR7142643	25.78

Table S12. Distribution of heterozygous SNPs in Ossabaw pig genome, Related to Figure 1.

	Intergenic	Intron	Exon
Number	2,610,736	1,431,912	33,079

Table S14. List of heterozygous genes related to fatty acid metabolism in Ossabaw pig genome, Related to Figure 1.

**Gene ID**	**Number of SNPs in exons**	**Description from KEGG database**
Ossabaw00174	10	MAP3K5; mitogen-activated protein kinase kinase kinase 5 isoform X1
Ossabaw00287	3	cytochrome c oxidase subunit 7C, mitochondrial;
Ossabaw00419	1	zinc-binding dehydrogenase family protein;
Ossabaw01443	1	ABHD17B; protein ABHD17B isoform X1
Ossabaw01550	5	ALDH1B1; aldehyde dehydrogenase X, mitochondrial
Ossabaw01589	29	acyl-coenzyme A amino acid N-acyltransferase 2;
Ossabaw01592	2	LOW QUALITY PROTEIN: bile acid-CoA:amino acid N-acyltransferase-like;
Ossabaw01603	2	CYCS; cytochrome c
Ossabaw01621	1	ABCA1; ATP-binding cassette sub-family A member 1
Ossabaw01623	11	ABCA1; ATP-binding cassette sub-family A member 1
Ossabaw01933	11	RXRA; retinoic acid receptor RXR-alpha isoform X1
Ossabaw02279	2	HACD1, PTPLA; 3-hydroxyacyl-CoA dehydratase 1
Ossabaw02295	2	OLAH; LOW QUALITY PROTEIN: S-acyl fatty acid synthase thioesterase, medium chain
Ossabaw02795	3	IRS2; LOW QUALITY PROTEIN: insulin receptor substrate 2
Ossabaw03020	5	ERN1; serine/threonine-protein kinase/endoribonuclease IRE1
Ossabaw03546	2	PROCA1; protein interacting with cyclin A1
Ossabaw03589	2	CYCS; cytochrome c
Ossabaw03848	5	ALDH3A2; fatty aldehyde dehydrogenase isoform X2
Ossabaw03875	9	SREBF1; sterol regulatory element-binding protein 1
Ossabaw04844	1	ADIPOQ; adiponectin precursor

Ossabaw05655	1	MLXIP; MLX-interacting protein isoform X1
Ossabaw05783	1	acetyl-CoA carboxylase-like;
Ossabaw06450	2	NDUFB8; NADH dehydrogenase [ubiquinone] 1 beta subcomplex subunit 8, mitochondrial
Ossabaw06553	11	CASP7, Caspase-7; caspase 7
Ossabaw06569	5	PNLIP; pancreatic triacylglycerol lipase precursor
Ossabaw06571	4	PNLIPRP2; pancreatic lipase-related protein 2 precursor
Ossabaw06624	5	ACADSB; short/branched chain specific acyl-CoA dehydrogenase, mitochondrial
Ossabaw07574	2	NDUFA10; NADH dehydrogenase [ubiquinone] 1 alpha subcomplex subunit 10, mitochondrial
Ossabaw07934	6	ADIPOQ; adiponectin, C1Q and collagen domain containing
Ossabaw08145	12	CYTB; cytochrome b
Ossabaw08832	1	NDUFB2; NADH dehydrogenase [ubiquinone] 1 beta subcomplex subunit 2, mitochondrial
Ossabaw08941	4	CYCS; cytochrome c
Ossabaw09264	2	CPT1A; carnitine O-palmitoyltransferase 1, liver isoform
Ossabaw10392	2	NDUFA7; NADH dehydrogenase [ubiquinone] 1 alpha subcomplex subunit 7
Ossabaw10433	2	cytochrome c-like; K08738 cytochrome c
Ossabaw10438	2	ACSBG2; long-chain-fatty-acid--CoA ligase ACSBG2
Ossabaw10631	1	cytochrome c-like; K08738 cytochrome c
Ossabaw10917	10	ALDH7A1; alpha-aminoadipic semialdehyde dehydrogenase
Ossabaw10922	1	putative acetyl-CoA acetyltransferase;
Ossabaw11199	9	GB18611; uncharacterized LOC726760
Ossabaw11523	5	cytochrome b-c1 complex subunit 2, mitochondrial; K00415 ubiquinol-cytochrome c reductase core subunit 2
Ossabaw11597	44	uncharacterized protein LOC110241526; K14462 apolipoprotein B
Ossabaw11865	5	acyl-CoA dehydrogenase; K00232 acyl-CoA oxidase [EC:1.3.3.6]
Ossabaw11979	1	EIF2AK3; eukaryotic translation initiation factor 2-alpha kinase 3
Ossabaw12861	2	succinate dehydrogenase [ubiquinone] flavoprotein subunit, mitochondrial;
Ossabaw13030	3	ALDH9A1; 4-trimethylaminobutyraldehyde dehydrogenase isoform X1
Ossabaw15113	8	cytochrome c oxidase subunit 6B1 isoform X2; K02267 cytochrome c oxidase subunit 6b
Ossabaw16234	1	MECR; trans-2-enoyl-CoA reductase, mitochondrial
Ossabaw16559	59	taurochenodeoxycholic 6 alpha-hydroxylase isoform X1;
Ossabaw16726	4	CPT2; carnitine O-palmitoyltransferase 2, mitochondrial precursor
Ossabaw16769	10	taurochenodeoxycholic 6 alpha-hydroxylase isoform X1;
Ossabaw16909	3	ECI2; enoyl-CoA delta isomerase 2, mitochondrial
Ossabaw17660	6	NDUFA4; cytochrome c oxidase subunit NDUFA4
Ossabaw18266	5	ACOX3; peroxisomal acyl-coenzyme A oxidase 3 isoform X1
Ossabaw19289	37	MOGAT2; 2-acylglycerol O-acyltransferase 2
Ossabaw19453	1	ACAT1; acetyl-CoA acetyltransferase, mitochondrial isoform X1

Table S16. Data information used in this research, Related to Figure 1 and Figure 2.

Species	NCBI accession
Human	GCF_000001405.39
Mouse	GCF_000001635.26
Dog	GCF_000002285.3
Horse	GCF_002863925.1
Camel	GCF_000803125.2
Goat	GCF_001704415.1
Cattle	GCF_002263795.1
Duroc	GCF_000003025.6
opossum	GCF_000002295.2
Bama_miniature	GCA_007644095.1
Bamei	GCA_001700235.1
Hampshire	GCA_001700165.1
Jinhua	GCA_001700295.1
Landrace	GCA_001700215.1
LargeWhite	GCA_001700135.1
Meishan	GCA_001700195.1
Pietrain	GCA_001700255.1
Rongchang	GCA_001700155.1
Tibetan	GCA_000472085.2
Wuzhishan	GCA_000325925.2

Table S18. SNPs distribution on each chromosome of the Ossabaw genome, Related to Figure 3.

ID	Total	Intergenic	Intron	Exon
chromosome_1	473,769	311,258	159,939	2,617
chromosome_10	167,754	95,443	71,602	844
chromosome_11	76,743	53,575	23,002	166
chromosome_12	116,308	61,149	53,706	1,565
chromosome_13	265,219	184,850	79,364	1,051
chromosome_14	296,218	175,999	118,637	1,889
chromosome_15	135,355	95,654	39,032	671
chromosome_16	129,810	96,163	33,370	277
chromosome_17	133,904	74,158	58,923	835
chromosome_18	114,349	63,493	50,081	775
chromosome_2	271,177	175,442	91,760	4,255
chromosome_3	262,940	146,465	114,374	2,328
chromosome_4	210,569	134,870	74,458	1,275
chromosome_5	142,822	97,503	44,379	993
chromosome_6	360,360	215,470	141,952	3,080

chromosome_7	162,343	102,515	58,036	1,831
chromosome_8	267,747	167,932	98,799	1,134
chromosome_9	165,548	112,351	51,685	1,531
chromosome_X	54,350	34,965	19,042	370

Table S20. Eight high density regions larger than 5 Mb with consecutive 500 kb bins, Related to Figure 3.

**Chr**	**Start**	**End**
1	235,500,001	247,000,000
12	42,000,001	48,000,000
14	106,500,001	115,500,000
2	60,500,001	69,000,000
2	72,500,001	82,000,000
2	500,001	8,500,000
6	80,500,000	86,500,000
7	19,500,001	25,500,000

Table S22. Obesity related to genes with indels in exons, Related to Figure 3.

Gene ID	Gene symbol
NP_001159788.1	MNAT1
XP_020919517.1	KDM4C
NP_001026948.1	PLIN3
NP_001106758.1	LDHB
NP_001243699.1	NLRP3
XP_020938133.1	MUC6
XP_020938242.1	MUC5AC
XP_020938243.1	MUC2
XP_020939610.1	ADGRE1
XP_020939713.1	PLIN4
XP_020941596.1	PRKAR1B
XP_020942837.1	REV1
XP_020943636.1	MUC12
XP_003125633.3	CNGB3
XP_003361737.2	COLEC10
XP_020945388.1	MUC1
XP_020946053.1	PLEC
XP_005664259.1	MYBPC1
XP_020947029.1	CELSR1
XP_020948723.1	PTPRQ
XP_020950053.1	SPTBN4

XP_020950146.1	ZNF283
XP_013852615.1	MESP2
XP_020953956.1	ACAN
XP_020954735.1	CD276
XP_020956418.1	HERC6
XP_020957142.1	FGA
XP_020957459.1	GPAT3
XP_005667607.2	C4BPA
XP_020919077.1	ABCB5
XP_020919318.1	TNN
XP_020919401.1	TDRD5
XP_020957820.1	CD55
XP_005668066.2	CYB5R1
XP_020921072.1	CENPJ
XP_020921243.1	SUCLA2
XP_020921581.1	ARHGEF7
XP_020921664.1	PARP4
XP_003131508.1	KRT10
XP_005656960.2	CYTH1
XP_020923541.1	DNAH9
XP_020923931.1	MUC4
XP_003483459.2	UBC
XP_005670496.2	NEFM
XP_005670835.2	NEFH
XP_005671024.1	TARBP1
XP_020928723.1	RP1L1
XP_020929397.1	MKI67
XP_020930397.1	PLCD4
XP_005672486.2	RICTOR
XP_020932479.1	NAIP
XP_020933695.1	RRBP1
XP_020937495.1	FMN2
NP_999164.2	DUOX2
NP_001116460.1	HMGCR
XP_020946919.1	EEA1
XP_020949331.1	CA5A
XP_005663896.2	APOL3

Table S23. Gene expression (FPKM) of four pseudogenes (Walker et al. 2019), Related to Figure 3.

Genes	WD -1	WD -2	WD -3	WD -4	WD -5	WD -6	WD -7	HHD -1	HHD -2	HHD -3	HHD -4	HHD -5	HHD -6

NSUN6	0	0	0	1.77	1.42	0	0	2.3	0	0	0	0	0
RAB18	0	0	0	0	0	0	0	0	0	0	1.95	0	0
SLC39A2	0	0	0	0	0	0	0.28	0	0.12	0	0.1	0	0
CUBN	0	0	0.03	0	0.1	0	0	0	0	0.11	0	0	0.29

Table S24. Unique gene families in Ossabaw genome with KEGG annotation, Related to Figure 4.

Gne KO ID (KEGG)	Copy number	Description
K00522	2	ferritin heavy chain-like
K00573	157	PCMT1; protein-L-isoaspartate (D-aspartate) O-methyltransferase
K01142	39	hypothetical protein; exodeoxyribonuclease III
K01229	3	LCT; lactase; K01229 lactase-phlorizin hydrolase
K01495	3	GCH1; GTP cyclohydrolase 1
K01530	1	probable phospholipid-transporting ATPase IH
K02737	2	Psmb5; proteasome subunit beta 5
K02910	17	uncharacterized LOC103555032; large subunit ribosomal protein L31e
K03231	3	elongation factor 1-alpha 1-like
K04257	43	olfactory receptor-like
K04534	6	GNAO1; G protein subunit alpha o1
K04810	1	CHRNA9; cholinergic receptor nicotinic alpha 9 subunit
K04953	36	CNGB3; cyclic nucleotide-gated cation channel beta-3
K04985	70	PKD1; polycystin 1, transient receptor potential channel interacting
K05030	8	epithelial chloride channel protein-like
K05620	1	protein-glutamine gamma-glutamyltransferase E-like
K05673	2	multidrug resistance-associated protein 4-like
K06464	1	integrin beta-2-like
K06553	1	immunoglobulin lambda-like polypeptide 5
K06554	1	IGLL1; immunoglobulin lambda-like polypeptide 1 precursor
K06634	3	CCNH, Cyclin-H; cyclin H
K08742	3	E3 ubiquitin-protein ligase SIAH2
K08857	1	NEK4; serine/threonine-protein kinase Nek4
K09228	12	uncharacterized LOC101935088; KRAB domain-containing zinc finger protein
K10408	11	dynein heavy chain 11, axonemal
K10730	2	hypothetical protein; ATP-dependent DNA helicase Q4
K10798	89	poly [ADP-ribose] polymerase 4-like
K11290	32	LRRGT00152; similar to Set alpha
K12321	1	olfactory guanylyl cyclase GC-D-like
K12322	1	GUCY2F; LOW QUALITY PROTEIN: retinal guanylyl cyclase 2
K12678	1	hypothetical protein; autotransporter family porin

K13624	5	ceruloplasmin-like
K14861	1	URB1; URB1 ribosome biogenesis 1 homolog
K17495	22	CUB and sushi domain-containing protein 1-like
K17503	4	PPM1H; protein phosphatase, Mg2+/Mn2+ dependent 1H
K17784	2	MINOS1; MICOS complex subunit MIC10 isoform X1
K18080	2	tensin-3
K18710	1	histone RNA hairpin-binding protein-like
K19464	3	hypothetical protein;pleckstrin homology domain-containing family G member 5
K19924	1	SPINK13; serine protease inhibitor Kazal-type 13
K19941	37	uncharacterized LOC102351696; synapsin
K20217	3	ubiquitin-conjugating enzyme E2 E2
K21449	1	large adhesin
K21774	2	Lin37, 1810054G18Rik; lin-37 homolog
